# Investigation into the mechanism of action of the antimicrobial peptide epilancin 15X

**DOI:** 10.3389/fmicb.2023.1247222

**Published:** 2023-11-02

**Authors:** Chunyu Wu, B. Alexis Lower, Ryan Moreira, Darian Dorantes, Tung Le, Constantin Giurgiu, Yanxiang Shi, Wilfred A. van der Donk

**Affiliations:** ^1^Department of Biochemistry, University of Illinois at Urbana−Champaign, Champaign, IL, United States; ^2^Department of Chemistry, The Howard Hughes Medical Institute, University of Illinois at Urbana−Champaign, Champaign, IL, United States

**Keywords:** lanthipeptide, antibiotic, *Staphylococcus*, membrane-active, microbiome, RiPPs

## Abstract

Addressing the current antibiotic-resistance challenge would be aided by the identification of compounds with novel mechanisms of action. Epilancin 15X, a lantibiotic produced by *Staphylococcus epidermidis* 15 × 154, displays antimicrobial activity in the submicromolar range against a subset of pathogenic Gram-positive bacteria. *S. epidermidis* is a common member of the human skin or mucosal microbiota. We here investigated the mechanism of action of epilancin 15X. The compound is bactericidal against *Staphylococcus carnosus* as well as *Bacillus subtilis* and appears to kill these bacteria by membrane disruption. Structure–activity relationship studies using engineered analogs show that its conserved positively charged residues and dehydroamino acids are important for bioactivity, but the N-terminal lactyl group is tolerant of changes. Epilancin 15X treatment negatively affects fatty acid synthesis, RNA translation, and DNA replication and transcription without affecting cell wall biosynthesis. The compound appears localized to the surface of bacteria and is most potent in disrupting the membranes of liposomes composed of negatively charged membrane lipids in a lipid II independent manner. Epilancin 15X does not elicit a LiaRS response in *B. subtilis* but did upregulate VraRS in *S. carnosus*. Treatment of *S. carnosus* or *B. subtilis* with epilancin 15X resulted in an aggregation phenotype in microscopy experiments. Collectively these studies provide new information on epilancin 15X activity.

## 1. Introduction

Antimicrobial resistance is a present-day global threat (Dadgostar, 2019). A rising concern for antimicrobial resistance is the limited number of targets affected by our current pool of antibiotics. Over two hundred approved antibacterial drugs affect a small number of bacterial processes, including DNA replication, RNA synthesis, protein synthesis, folate synthesis, and cell wall biosynthesis (Cotsonas King and Wu, 2009). In recent years, an increasing number of pathogenic bacteria have overcome the action of these antibiotics by developing drug-resistant mutations in the original targets or developing alternative resistance mechanisms. To combat this issue, the discovery of antibacterial agents that function through novel modes of action is important.

Ribosomally synthesized and post-translationally modified peptides (RiPPs) are a large class of natural products that have received increasing attention in recent years. The extensive post-translational modifications (PTMs) endow RiPPs with remarkable structural diversity and an associated wide range of biological activities from antimicrobial to antiallodynic (Arnison et al., 2013; Li and Rebuffat, 2020; Cao et al., 2021; Ongpipattanakul et al., 2022). Lanthipeptides are a large subfamily of RiPPs that contain thioether crosslinked amino acids (Repka et al., 2017). When they display antimicrobial activity, lanthipeptides are called lantibiotics. The mechanisms by which these compounds exert their antibacterial activity have been studied extensively for a subset of members, with nisin as the best-understood example (Breukink and de Kruijff, 2006; Wang et al., 2020; Ongpipattanakul et al., 2022). Lipid II and phosphatidylethanolamine (PE) are the only two direct lantibiotic targets that have been identified thus far (Märki et al., 1991; Brötz et al., 1997, 1998; Breukink et al., 1999; Breukink and de Kruijff, 2006; Wiedemann et al., 2006; Smith et al., 2008; Oman et al., 2011; Munch et al., 2014; Fujinami et al., 2018; Ongpipattanakul et al., 2022; Buijs et al., 2023; Deisinger et al., 2023). It is intriguing that both of these targets are non-macromolecular (i.e., not DNA, RNA, or protein), which provides challenges for the development of resistance since target structure alteration is more complicated than in the case of macromolecular targets for which mutation is sufficient to change structure (Breukink and de Kruijff, 1999; Lewis, 2020). Therefore, identifying targets of additional lantibiotics is a promising area of research.

**Figure 1:**
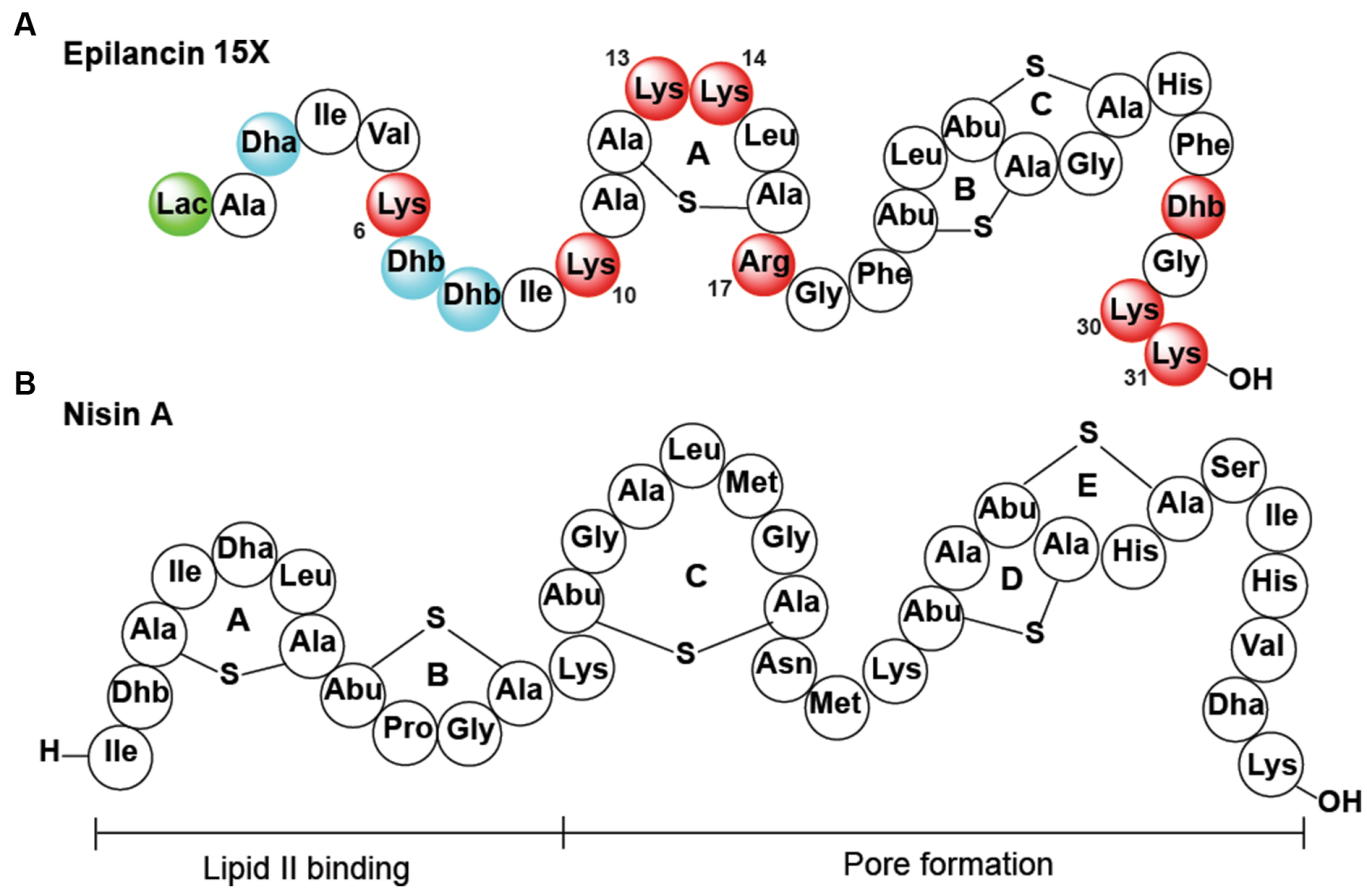
**(A)** Epilancin 15X structure. Substitution of the residues in red resulted in reduction/loss of antibacterial activity, simultaneous substitution of the residues in blue was deleterious for antibacterial activity, and replacement of the residue in green did not result in a change in antibacterial activity. Residues that are not colored were not assessed. Lac, lactate. **(B)** Nisin A structure.

Epilancin 15X was isolated from the clinical isolate *Staphylococcus epidermidis* 15 × 154. The compound is a member of the epilancin-group of lanthipeptides that has potent antimicrobial activity against several pathogenic bacteria, including methicillin-resistant *Staphylococcus aureus* (MRSA) and vancomycin-resistant enterococci (VRE), with minimal inhibitory concentrations (MICs) in the nanomolar range (van de Kamp et al., 1995a; Ekkelenkamp et al., 2005; Wang et al., 2023). Epilancin 15X contains an unusual N-terminal 2-hydroxypropionyl group (lactate, Lac), one lanthionine (Lan) bridge, and two intertwined methyllanthionine (MeLan) rings on the C-terminus of the polypeptide (Figure 1A; Ekkelenkamp et al., 2005). The epilancin 15X biosynthetic gene cluster contains five genes involved in its biosynthesis (*elxABCOP*; Velásquez et al., 2011). The precursor peptide ElxA is modified by the glutamyl-tRNA dependent dehydratase ElxB that dehydrates Ser and Thr residues to dehydroalanine (Dha) and dehydrobutyrine (Dhb) residues, respectively. This step is followed by thioether formation by the cyclase ElxC that generates Lan and MeLan crosslinks (Velásquez et al., 2011). Next, the protease ElxP removes a leader peptide, and the dehydrogenase ElxO converts an N-terminal pyruvyl group (generated by hydrolysis of an N-terminal Dha) to lactate to form the mature epilancin 15X (Velásquez et al., 2011).

The details of the mode of action of epilancin 15X remain to be elucidated (Wang et al., 2023). The best-studied mechanism of lantibiotic action is that of nisin, which inhibits bacterial growth by binding to the pyrophosphate moiety of lipid II with its A and B rings (Breukink et al., 1999; Hsu et al., 2004). Lipid II binding leads to the inhibition of the transglycosylation reaction necessary to synthesize cell wall peptidoglycan (Wiedemann et al., 2001, 2004; Hasper et al., 2006). Upon binding to lipid II, the D and E rings of nisin are believed to insert and form pores in the membrane, leading to bacterial cell death (Wiedemann et al., 2001; Hasper et al., 2004; Hsu et al., 2004; Wiedemann et al., 2004). From a structural perspective, epilancin 15X is missing the A and B rings of nisin that bind lipid II (Figures 1A,B), and indeed previous experiments have suggested the molecule (or its structural analog epilancin K7) does not bind to lipid II *in vitro* (Brötz et al., 1998; Oman et al., 2011). But the B and C rings of epilancin 15X are similar to the D and E rings of nisin that are believed to be involved in pore formation (van Kraaij et al., 1998), and lipid II was shown to antagonize epilancin 15X action (Wang et al., 2020). The formation of pores alone, however, does not appear to explain the low MIC values of epilancin 15X against staphylococci as most cationic pore-forming peptides have MIC values in the micromolar range (Hoffmann et al., 2004). In the case of nisin, its potent nanomolar activity is a consequence of docking onto the target lipid II, which not only inhibits cell wall biosynthesis but also facilitates formation of pores that are composed of lipid II and nisin (Breukink et al., 1999, 2003; Hasper et al., 2004; Hsu et al., 2004). With epilancin 15X lacking the lipid-II binding motif, it raises the question how epilancin 15X achieves its high potency against select bacteria. A very recent study showed that the compound is bacteriostatic against some bacteria, whereas bactericidal activity was observed with other bacteria, and that the latter activity coincides with pore formation (Wang et al., 2023).

Epilancin 15X is part of a group of lantibiotics that recently were proposed to potentially utilize a centrally located five-amino acid ring (A-ring in epilancin 15X; Figure 1) containing a positively charged residue in the second position of the ring (Lys13 in epilancin 15X) and two positively charged residues up and downstream of this ring (Lys10 and Arg 17 in epilancin 15X; Wang et al., 2020). This structural motif is found in all known non-lipid II targeting lantibiotics (members of the epilancin, Pep5, and paenibacillin groups that are all highly cationic), but the functional importance of the motif has not yet been investigated.

To study the molecular mechanism of epilancin 15X, we conducted in this study structure–activity relationship (SAR) studies of epilancin 15X to assess the importance of the N-terminal lactate group, the dehydrated amino acids residues, and the positively charged residues through generating epilancin 15X analogs in *Escherichia coli*. Additionally, the incorporation of radioactive precursors involved in cell wall, DNA, RNA, and protein synthesis in *S. carnosus* TM300 was monitored upon epilancin 15X treatment. Using membrane permeability studies, we show that epilancin 15X disrupts the integrity of the bacterial membrane in *S. carnosus* TM300 and *B. subtilis* BSF2470. Consistent with these results a fluorescently labeled analog was localized in a punctate pattern on the cell surface. A series of experiments provide evidence against the involvement of lipid II as a direct target, but liposome leakage assays showed that negatively charged membrane lipids are important for epilancin 15X activity.

## 2. Materials and methods

### 2.1. General methods

Oligonucleotides and enzymes were purchased from Integrated DNA Technologies (IDT) and New England Biolabs (NEB), respectively. Polymerase chain reaction (PCR) amplifications were carried out using Q5 polymerase (NEB) on an automated thermocycler (C1000, Bio-Rad). DNA sequencing was performed using appropriate primers from ACGT, Inc. Matrix-assisted laser desorption/ionization time-of-flight mass spectrometry (MALDI-TOF MS) analyses were conducted at the Mass Spectrometry Facility at UIUC using a Bruker UltrafleXtreme MALDI TOF/TOF spectrometer (Bruker Daltonics). For MALDI−TOF MS analysis, samples were desalted using ZipTipC18 (Millipore) and spotted onto a MALDI target plate with a matrix solution consisting of a saturated aqueous solution of super DHB (2,5-dihydroxy benzoic acid; Sigma-Aldrich). Peptides were purified by reversed-phase high performance liquid chromatography (RP − HPLC) on an Agilent 1,260 Infinity II instrument equipped with a Macherey-Nagel C18 reversed-phase column (4.6 mm i.d. × 250 mm L). For RP − HPLC, solvent A was 0.1% trifluoroacetic acid (TFA) in H_2_O, and solvent B was acetonitrile containing 0.1% TFA. An elution gradient from 0% solvent B to 100% solvent B over 30 min was used unless specified otherwise with a flow rate of 1 mL/min.

### 2.2. Materials and bacterial growth conditions

Chemical reagents and media components used in this study were purchased from Sigma-Aldrich or Thermo Fisher Scientific, unless otherwise specified. The strains, primers and DNA gene fragments are listed in Supplementary Tables S1, S2. *Staphylococcus* strains were grown in brain-heart infusion (BHI) liquid broth at 37°C. *Bacillus* strains and *E. coli* strains were grown in Luria-Bertani (LB) broth at 37°C. *S. carnosus* TM300 was a gift from Prof. Gabriele Bierbaum (Bonn University); *B. subtilis* BSF2470 (1A980) was obtained from The Bacillus Genetic Stock Center (Columbus, Ohio). The epilancin producer *S. epidermidis* 15 × 154 was a gift from Prof. Jan Verhoef (Utrecht University Hospital).

### 2.3. Cloning of epilancin 15X analogs

Plasmid assembly and propagation were conducted in NEB Turbo cells (New England Biolabs). Expression of recombinant peptides and proteins was performed after transformation of chemically competent *E. coli* BL21 (DE3) cells (New England Biolabs) with the appropriate plasmid. Single-stranded DNA primers and double-stranded DNA gene fragments were purchased from Integrated DNA Technologies. All ElxA inserts were placed in between digestion sites BglII and NotI on a pET21a plasmid. The gene encoding each mutant ElxA was purchased as a double-stranded DNA fragment (IDT) and assembled into the pET21a plasmid (Supplementary Table S2). The plasmid pET21a was linearized by PCR using Q5 master mix (New England Biolabs). The PCR products were purified by QIAprep spin column (Qiagen) and the concentration of DNA was quantified by Nanodrop absorbance (Thermo Fisher). The linear DNA fragments were mixed at 1:5 molar ratio (backbone to insert) and assembled using *E. coli* NEB HiFi Master Mix at 50°C for 1 h. The assembly reaction was used to transform chemically competent NEB Turbo cells and plated on LB agar with ampicillin (100 μg/mL). All plasmids were confirmed by DNA sequencing through ACGT, Inc.

### 2.4. Heterologous expression of epilancin 15X and its analogs in *Escherichia coli*

Isolation of wild-type epilancin 15X from the producer strain was performed as described previously (Velásquez et al., 2011) resulting in 10 mg per liter of culture. The heterologous expression in *E. coli* and purification of epilancin 15X was performed as reported previously (Lee et al., 2023). The gene encoding each mutant precursor peptide was purchased as a double-stranded DNA fragment (IDT) and assembled into the pET21a vector. The assembled vector carrying His_6_-ElxA was co-expressed with pRSF_elxB.elxC and pEVOL_GluRS.tRNA as reported previously (Lee et al., 2023). The purified His_6_-ElxA was treated with purified ElxP protease to liberate the mature modified core peptide (Velásquez et al., 2011). Since ElxO was not added, all products contain a pyruvyl group instead of a lactyl group on the N-terminus (Velásquez et al., 2011). The fully dehydrated modified core peptide was further purified using RP-HPLC and analyzed by MALDI-TOF MS. It was recently reported that it is essential to remove trifluoroacetate from HPLC-purified epilancin 15X for maximum bioactivity (Wang et al., 2020). Indeed, we confirmed that such removal greatly decreased MIC values. To remove TFA from HPLC-purified peptides, 2 mM HCl was added to the peptide. The peptide solution was frozen with liquid nitrogen and lyophilized overnight to remove all liquid. The peptide was redissolved in 2 mM HCl and the lyophilization process was repeated at least two times. After a final lyophilization step, the peptide was dissolved in water to the desired concentration. A total of 0.3 mg of fully dehydrated core peptide was obtained per liter of *E. coli*.

### 2.5. MIC determination and agar diffusion bioactivity assays

MIC determination was performed in a 96-well microtiter plate as described previously (Wu et al., 2019). Sensitive bacterial culture was grown in the appropriate medium (BHI for *S. carnosus*; LB for *B. subtilis*) at 37°C overnight. The culture was diluted to 10^5^ CFU/mL in medium in each well. Epilancin 15X or its analog was dissolved in water and serially diluted to obtain final concentrations of 20, 10, 5, 2.5, 1.25, 0.625, 0.3125, 0.156, 0.078, 0.039, 0.019, and 0.009 μM. OD_600_ readings were obtained after 18 h using a Synergy H4 Hybrid Multi-Mode Microplate Reader (BioTeK). Cell-free untreated medium, and untreated cell culture were used as negative controls. The MIC of nisin against *B. subtilis* BSF2470 was determined following the same methods.

For agar diffusion antimicrobial activity assays, the autoclaved 1.5% agar was prepared in the appropriate medium and cooled in a 40°C water bath for 15 min. A total of 50 μL overnight culture of the sensitive strain was added to the agar and kept in the water bath. A total of 25 mL seeded agar was poured into a sterile OmniTray (Nunc) and allowed to solidify at room temperature for 30 min. An additional 35 mL of seeded agar was poured over the lower solidified agar layer. A sterile 96-well PCR plate was placed in the molten agar upper layer, and the agar was allowed to solidify at 25°C for 30 min. After sufficient solidification, the 96-well PCR plate was removed. Then 20 μL volume of each peptide was dispensed into the wells. Plates were left at 25°C for 30 min until the wells were dried, and the plates were incubated at 37°C overnight. The antibacterial activity was determined by the presence or absence of growth inhibition.

### 2.6. Epilancin 15X killing kinetics against *Staphylococcus carnosus* and *Bacillus subtilis*

*S. carnosus* TM300 and *B. subtilis* BSF2470 (1A980) cell cultures were grown to mid-log phase in BHI and LB broth, respectively. Epilancin 15X at 1x and 4x MIC (0.15 μM for 1x MIC in *S. carnosus*; 2.5 μM for 1x MIC in *B. subtilis*) were added to the cell culture and incubated for 30 min to 180 min. At each time point, an aliquot of 5 mL treated-culture was serial-diluted and placed on BHI agar plates for *S. carnosus* and LB agar plates for *B. subtilis*. The plates were grown for 1 day at 37°C and the colonies were counted. Each experiment was performed in triplicate and untreated cell culture was used as the negative control.

### 2.7. Membrane permeability study with propidium iodide

Cultures of *S. carnosus* TM300 and *B. subtilis* BSF2470 were grown to OD_600_ 1.0 and diluted to OD_600_ 0.1 in fresh BHI or LB medium, respectively. The culture was then combined with a final concentration of 25 μM PI (ThermoFisher P3566), 1 mM HEPES, and 1 mM glucose. Next, epilancin 15X (0.5x MIC, 1x MIC, and 4x MIC; 1x MIC 0.15 μM for *S. carnosus*; 1x MIC 2.5 μM for *B. subtilis*) or nisin (0.5x MIC, 1x MIC, and 4x MIC; 1x MIC 0.5 μM for *S. carnosus*; 0.625 μM for *B. subtilis*) was added and the cells were incubated for 20 min at room temperature before analysis. Another two tubes were prepared without antibiotic treatment as controls. The culture was analyzed with a BD Biosciences LSR II flow cytometer for *S. carnosus* and a BD Biosciences LSRFortessa for *B. subtilis* using excitation at 488 nm with an argon laser and measurement of emission through a band-pass filter at 695/40 nm. A minimum of 30,000 events were detected for each sample. The experiment was repeated three times, and the mean PI uptake value was determined. The results were calibrated such that the mean PI uptake from the 4x MIC nisin-treated cell culture was set as 100% PI uptake.

### 2.8. Membrane potential studies

Cultures of *S. carnosus* TM300 were grown to OD_600_ 1.0 and diluted to OD_600_ 0.1 in fresh BHI medium. The culture was then combined with 30 μM 3,3′-diethyloxacarbocyanine (DiOC2, ThermoFisher D14730) and incubated for 20 min. Then epilancin 15X (0.5x MIC, 1x MIC, and 4x MIC; 1x MIC 0.15 μM) or nisin (0.5x MIC, 1x MIC, and 4x MIC; 1x MIC 0.5 μM) was added and the cells were incubated for another 20 min at room temperature before analysis. Another two tubes were prepared without antibiotic treatment as controls. The culture was analyzed with a BD Biosciences LSR II flow cytometer using excitation at 488 nm with an argon laser and measurement of emission through a band-pass filter at 616/20 nm. A minimum of 30,000 events were detected for each sample. The experiment was repeated three times, and the mean red/green fluorescence ratio was determined.

### 2.9. Macromolecular synthesis assays

Radiolabel incorporation experiments were carried out similarly as previously described but using *S. carnosus* (Wu et al., 2019). The OD_600_ of parallel nonradioactive cultures was measured at each time point to normalize the measured radioactivity and minimize effects due to cell death and growth inhibition. The radiolabeled reagents and their corresponding positive and negative antibiotic controls were as follows. Fatty acid: [^14^C]-acetic acid (American Radiolabeled Chemicals, ARC 0101A), positive control: isoniazid (Sigma), negative control: ciprofloxacin. Cell wall: [^14^C]-N-acetyl-D-glucosamine (American Radiolabeled Chemicals, ARC 0105), positive control: vancomycin, negative control: ciprofloxacin. DNA: [^14^C]-thymidine (American Radiolabeled Chemicals, ARC 1219), positive control: ciprofloxacin, negative control: rifampicin. RNA: [5-^3^H]-uridine (PerkinElmer, NET174250UC), positive control: rifampicin, negative control: ciprofloxacin. Amino acids: [^14^C]-L-amino acid mixture (PerkinElmer, NEC850E050UC), positive control: chloramphenicol, negative control: ciprofloxacin. All experiments were conducted independently three times.

### 2.10. Cytochrome c binding assay

This procedure is based on one previously reported (Mukhopadhyay et al., 2007). Briefly, cells were grown overnight in the appropriate medium at 37°C. Cells were collected by centrifugation at 2800 ×g for 10 min. The cell pellets were washed with MOPS buffer (20 mM, pH = 7), then resuspended in MOPS buffer, pelleted, and washed again. Cells were resuspended in the same buffer to achieve an OD_578_ of 7. Cytochrome c was then added to the cell suspension to achieve a concentration of 0.5 mg/mL and this mixture was incubated for 10 min. Afterwards, the cell suspension was pelleted by centrifugation (2,800 ×g for 10 min) and the absorbance of the supernatant at 530 nm was determined. The fraction of cytochrome c bound to the cells was determined by comparing the absorbance of each sample to the absorbance of the cytochrome c stock diluted to 0.5 mg/mL in cell-free MOPS buffer.

### 2.11. LiaRS response to epilancin 15X in *Bacillus subtilis* and RNAseq in *Staphylococcus carnosus*

The LiaRS response was determined via disk diffusion as described previously (Mascher et al., 2004). Briefly, *B. subtilis* strain BSF2470 was grown in LB supplemented with erythromycin (1 μg/mL) overnight at 37°C with shaking. A total of 20 μL of overnight culture was mixed with 100 μL of 20 mg/mL X-Gal (dissolved in DMF) and 5 mL of 0.7% soft LB agar then poured onto a 1.5% LB agar base. The plates were allowed to cool and dry at room temperature for 20 min. The desired molecule was either spotted on a filter paper disk and placed on top of the agar or directly spotted on the agar. The following molecules were tested: ampicillin, 0.5 μL on filter disk, 1 μL directly spotted, 100 mg/mL; compound 1771/LtaS-IN-1, 4 μL, 20 mM (dissolved in DMSO); epilancin 15X, 4 μL, 5 mM; vancomycin, 1 μL, 100 mg/mL; nisin, 4 μL, 2 mM; bacitracin, 4 μL on filter disk, 5 μL directly spotted, 100 mg/mL; water, 5 μL; magainin 2, 10 μL, 1 mM; melittin, 10 μL, 1 mM. The plate was incubated at 37°C overnight. After overnight incubation, the plate was imaged. Compound 1771 (CS-0114233) was purchased from ChemScene LLC.

For the RNAseq experiments, epilancin was added at sub-lethal concentration (0.5x MIC) to *S. carnosus* TM300 once the cultures reached an OD_600_ of 0.6. RNA was extracted 40 min after the treatment, using a FastRNA Pro Blue kit (MP Biomedicals). Both treated and parallel untreated samples were collected at the same time. Sequencing was performed at the Carver Biotechnology Center (UIUC). For RNAseq analysis, gene counts were read in to R (v3.1.1; Team, 2014), and 14/2441 genes were removed because they had fewer than 32 total counts across the six samples. edgeR’s (v3.6.7) TMM normalization (Robinson and Oshlack, 2010) plus negative binomial generalized log-linear model (McCarthy et al., 2012) was used to test for differential expression between treated and untreated groups, while accounting for pairing of U and T replicates from three different cultures. *p*-values were adjusted using the false discovery rate method (Benjamini and Hochberg, 1995).

### 2.12. Synthesis of epilancin-TAMRA

HPLC-purified epilancin 15X was dissolved in water at an initial concentration of 3.2 mM. A total of 1 mg TAMRA-thiol (BioActs) was dissolved in 40 μL of DMF to make a 50 mM stock. In a light-protected Eppendorf tube, 2 μL of 3.2 mM epilancin 15X was adjusted to pH 8.0 before the addition of 3 μL TAMRA-thiol. The reaction mixture was centrifuged briefly to collect the sample in the bottom of the tube and incubated at 25°C for 15 h protected from light. On the following day, the mixture was purified using an Agilent 1,260 Infinity II HPLC system equipped with a Macherey-Nagel C18 column (250 mm × 4.6 mm; catalog no.760002.46). Water containing 0.1% TFA was used as solvent A, while solvent B was acetonitrile containing 0.1% TFA. An elution gradient from 0% solvent B to 100% solvent B over 30 min at a flow rate of 1 mL/min was used, and epilancin 15X labeled with TAMRA was eluted at 55–60% solvent B. Fluorescent fractions were pooled and lyophilized.

### 2.13. Fluorescence and bright field microscopy

Standard microscope slides coated with poly-D-lysine (R&D Systems, 34–392 − 000101) overnight were washed with water and sterilized in ethanol. Overnight bacterial culture was centrifuged at 4,500 ×g and the pellet was resuspended gently three times in fresh PBS to remove the media background. Then 20 μL of cell culture in PBS and 180 μL of PBS were loaded on each slide and incubated at room temperature for 30 min. Next, 1 mL of PBS was used to wash the slides at least five times to remove unadhered cells. A final concentration of 200 nM epilancin 15X-TAMRA in 200 μL of PBS was applied to each slide and incubated at room temperature for 30 min. PBS was used to wash the slides at least five times to wash away unbound Epilancin 15X. A final concentration of 10% formaldehyde (Thermo Scientific, PI28906) and 2.5% glutaraldehyde (Fisher Chemical, O2957–1) in PBS was applied to each slide and incubated for 30 min. Three times 1 mL of PBS was applied to wash away excess fixing solution. A drop of ProLong Glass Antifade Mountant (Invitrogen, P36982) was applied to each slide, covered with a glass coverslip (Ibidi, NC0601315), and dried overnight protected from light. All micrographs were captured using a Zeiss LSM 880 Airyscan microscope (Carl R. Woese Institute for Genomic Biology, UIUC). The 63× phase-contrast objective (oil immersion) was used for image capture, in combination with ZEN 2.6 software (Zeiss) to control the microscope setup and to perform the imaging of cells. The visible excitation lines at 561 nm were used to visualize TAMRA.

### 2.14. Liposome permeabilization assay

For unilamellar liposome preparation, a total of 100 μL of 30 mM 1,2-dioleoyl-sn-glycero-3-phosphocholine (DOPC; Avanti Polar Lipids) and the following lipids dissolved in chloroform were mixed in a glass tube in the appropriate ratio: POPG (Avanti Polar Lipids), POPE (Avanti Polar Lipids), POPS (Avanti Polar Lipids), and purified *S. aureus* LTA (InvivoGen, catalog # tlrl-pslta). The solvent was evaporated under a stream of nitrogen gas and the glass vial was placed in a vacuum desiccator overnight. A total of 1 mL HEPES-Buffered Saline (HBS) composed of 5 mM HEPES (pH 7.6) and 150 mM NaCl, and 30 mM sulforhodamine B (SRB) was added to each vial. The lipid solution was vortexed for 10 min and lipids were hydrated by five freeze–thaw cycles with liquid N_2_. The lipid solution was diluted with HBS buffer to 3 mL and the solution was passed 29 times through a mini extruder (Avanti Polar Lipids) with a filter of 0.1-μM pore diameter to form lipid vesicles of homogeneous size. The extruded solution was passed through Sephadex 50 resin to remove the free sulforhodamine B (liposomes eluted faster than the dye). The size of the liposomes was tested using dynamic light scattering (DLS, Malvern Zetasizer, Dr. Catherine J. Murphy lab, Department of Chemistry, UIUC) and analyzed using Zetasizer software. The DLS data quality was interpreted by the instrument through its embedded algorithm to confirm the data quality. The polydispersity index (PDI), which measures the size distributions was used to monitor the quality of the liposomes. The small PDI indices obtained from DLS readings indicate that the liposomes were monodisperse (Farkas and Kramar, 2021). The total lipid concentration was measured with NMR spectroscopy using a reported method (Hein et al., 2016). The vesicles were stored at 4°C and used within 1–2 days.

For the dye leakage assay, 100 μL SRB-loaded liposomes (80 μM lipid; 100–200 nm size) were placed in each well in a 96-well microplate. Fluorescence of SRB-loaded liposomes was monitored over time on a Synergy H4 Hybrid Multi-Mode Microplate Reader (BioTeK) using λ_Ex_ at 480 nm and λ_Em_ at 517 nm. To each well, a different concentration of peptides or 0.02% triton X-100 was added 5 min after adding liposomes. Liposomes supplied with water were treated as the negative control. The fluorescence emission was measured every 15 s for 2 h. Fluorescence counts were recorded as % leakage and plotted as a function of time. The concentration of epilancin 15X where the SRB leaked from the liposomes reached half of the triton X-100 leakage was recorded as C_1/2_, and C_1/2_ values were plotted against DOPC and supplemented lipid concentrations.

### 2.15. Lipid II synthesis

Enzymatic synthesis of lipid II was adapted from a previous study (Huang et al., 2014) using phosphorylated undecaprenol (11-P), uridine 5′-diphospho-N-acetylglucosamine (UDP-GlcNAc), and UDP-MurNAc-pentapeptide (MMP). UDP-GlcNAc was purchased from Sigma-Aldrich. The enzymes required for lipid II assembly, MurG and MraY, were purified as described below. For small scale reaction, 10 μL of 11-P (10 mM in MeOH) was dried under nitrogen and resuspended in 50 μL of 2X buffer (100 mM HEPES pH 8.0, 10 mM MgCl_2_, 1% Triton X-100). A total of 10 μL of 10 mM UDP-GlcNAc and 10 μL of 10 mM UMP was added, followed by 10 μL MurG-CHis (100 μM stock) and 20 μL His-MraY (36 μM stock). The reaction was incubated at RT for 2–4 h and monitored by thin-layered chromatography using CHCl_3_:MeOH:H_2_O:NH_3_ (90:50:10:1) as solvent and iodine as staining method. TLC showed 11-P (Rf 0.5) and lipid II (Rf 0.2). After 11-P was fully converted, the reaction was extracted with 2 volumes of BuOH:pyridinium acetate (2:1). The organic phase was washed twice with 1 volume of water. The butanol phase was evaporated, the residue dissolved in MeOH and injected onto a Hypersil Gold C8 column (250 × 4.6 mm, 5 μm, 100 Å) connected to an RP-HPLC system (Agilent 1,260 Infinity II) running at 1 mL/min using 20 mM NH_4_HCO_3_:MeOH (1:1) as solvent A and MeOH:IPA (8:2) as solvent B. The applied gradient was 0–10 min: 0–100% B, 10–15 min: 100% B. Lipid II eluted at ~12.5 min. Fractions were collected and lyophilized to dryness. The purity of the synthesized lipid II was checked with TLC and LC–MS.

For LC–MS, the sample was injected onto a Kinetex C8 column (150 × 2.1, mm 2.6 μm, 100 Å) connected to an LC–MS-qTOF system (Agilent Infinity 1,260 II – qTOF 6,545) running at 0.3 mL/min using 20 mM NH_4_OAc:MeOH (1:1) as solvent A and MeOH:IPA (8:2) as solvent B. The applied gradient was 0–3 min: 0–100% B, 3–7 min: 100% B. qTOF MS was run in negative mode, collecting centroid data (100–2000 m/z) at 5 scan/s. The parameters were: Gas temp (200°C); Gas flow (13 L/min), Nebulizer (35 psi); Sheath gas temp (350°C); Sheath gas glow (11 L/min), Vcap (3,500 V); Nozzle voltage (1,000 V); Fragmentor (125 V); Skimmer voltage (65 V).

### 2.16. Cloning, expression and purification of MurG and MraY for lipid II assembly

Cloning: *murG* and the pET-28 backbone were amplified by colony PCR from *E. coli* BL21(DE3) cells using primers listed in Supplementary Table S2. The construct was assembled using Gibson Assembly to yield pET28:MurG-CHis which encoded MurG with a C-terminal His_6_ tag. Plasmid pTrc33:His_10_-HyMraY encodes MraY with an N-terminal His_10_ tag.

Expression and Purification: Terrific broth (TB) media (with kanamycin 50 μg/mL or chloramphenicol 25 μg/mL when the corresponding plasmid was used) was used for culture. *Escherichia coli* BL21 C43(DE3) was transformed with the desired plasmid. Colonies from the overnight plate were inoculated into 5 mL of TB media, grown at 37°C for 6–8 h, then sub-cultured into 2 L of TB. When the OD_600_ reached 1–1.5, the cells were incubated on ice for 10 min, induced with 0.25 mM IPTG, then cultured at 18°C for 18–20 h.

Purification of MraY: The cell pellet was harvested and resuspended in lysis buffer (20 mM Tris, 150 mM NaCl, 5% glycerol, 1 mM MgCl_2_, pH 8.0) at 3–5 mL/g wet weight then stored at −70°C until use. Cell pellet was lysed by passing two times through a French Press homogenizer (15,000–20,000 psi). The lysate was centrifuged at 75,000 ×g for 1 h. The pellet was collected and resuspended in a similar volume of Buffer A (20 mM Tris, 300 mM NaCl, 5% glycerol, and 0.5% dodecylmaltoside, pH 8.0), then gently shaken at 4°C overnight. The suspension was centrifuged at 30,000 ×g for 20 min to remove insoluble debris. The supernatant was incubated with Cobalt-NTA resin pre-equilibrated with Buffer A for 1 h at 4°C. The resin was washed with Buffer A + 10 mM imidazole until A_280_ < 0.05, then eluted with 4–5 column volumes of Buffer B (20 mM Tris, 300 mM NaCl, 150 mM imidazole, 5% glycerol, and 0.5% dodecylmaltoside, pH 8.0). The buffer was exchanged to Buffer A using an Amicon 10 K MWCO ultrafiltration device, the protein was flash-frozen and stored at −70°C.

Purification of MurG: The cell pellet was suspended in lysis buffer (20 mM Tris, 150 mM NaCl, 5 mM MgCl_2_, 20 mM 2-mercaptoethanol, 3% (v/v) Triton X-100, EDTA free protease inhibitor, pH 8.0) at 5 mL/g and lysed by passing two times through a French Press homogenizer (15,000–20,000 psi). The lysate was gently stirred at 4°C for 2 h, diluted 2-fold with Buffer A (20 mM Tris, 150 mM NaCl, pH 8.0), then centrifuged at 12,000 ×g for 30 min to remove insoluble debris. The supernatant was collected and incubated with Cobalt resin pre-equilibrated with Buffer A for 1 h at 4°C. The resin was washed with Buffer A + 10 mM imidazole until A_280_ was less than 0.05, then eluted with 4–5 column volumes of Buffer B (20 mM Tris, 150 mM NaCl, 300 mM imidazole, pH 8.0). The buffer was exchanged to Storage Buffer (20 mM Tris, 150 mM NaCl, 10 mM EDTA, 1 mM DTT, pH 8.0) using an Amicon 10 K MWCO ultrafiltration device, the protein was flash-frozen and stored at −70°C.

### 2.17. Liposome leakage assay using flow cytometry with liposomes containing lipid II

The liposome leakage assay using flow cytometry was adapted from a previous study that utilized liposome display to evolve α-hemolysin (Fujii et al., 2014). Briefly, the liposomes were made to encapsulate HaloTag protein whereas AF488-labeled HaloTag ligand was introduced to the outer solution together with the tested peptide. Upon pore formation, the ligand can move into the liposomes and form a covalent linkage with the HaloTag, which prevents the outflux of the ligand due to the size of the protein, resulting in the accumulation of fluorescent signal inside the liposome vesicle. During flow cytometry analysis, the fluorophores were excited with a built-in blue laser and the emission was monitored via the AF488 channel (530/30).

Liposome preparation: To prepare the lipid phase, 5 mmol POPC +5 mmol cholesterol (Avanti; with or without 0.1% mol lipid II) was dissolved in 100 μL of chloroform. Then 1 mL of paraffin oil was added, vortexed and heated with an opened tube at 80°C for 1 h to evaporate chloroform.

All of the next steps were performed on ice or in a cold room. MBP-HaloTag protein was dissolved in the inner solution (50 mM HEPES-KOH pH 7.6, 20 mM Mg(OAc)_2_, 100 mM potassium glutamate, 200 mM sucrose) to 10 μM. Then 20 μL of HaloTag solution was added to 200 μL of lipid phase in a 3-mL glass tube, followed by vigorous vortexing for 30 s. The emulsion was rested on ice for 5 min, then carefully layered on top of 200 μL outer solution (50 mM HEPES-KOH pH 7.6, 20 mM Mg(OAc)_2_, 100 mM potassium glutamate, 200 mM glucose) in a microcentrifuge tube and allowed to stand for 5 min to equilibrate the interface. The tube was then centrifuged at 9,000 ×g for 30 min. A white pellet (liposome) was formed at the bottom aqueous phase. The extra phospholipid was observed at the oil–water interface. To collect the liposome pellet, a pipet tip with a wide-opening attached to a pipetman set to 100 μL was inserted through the residual oil–water interphase into the aqueous phase near the pellet. Gently drawing and expelling the liquid in and out of the pipet tip detached the liposome pellet from the wall and drew it into the pipet tip. The pellet was gently resuspended and transferred to a new tube, followed by centrifugation at 9,000 ×g for 10 min. The supernatant was removed, and the liposome pellet was gently resuspended in 200 μL of inner solution.

Antibiotic testing by flow cytometry: The 25 μL of liposome solution prepared above was mixed with 25 μL of 2X inner buffer mix containing 100 nM AF488 HaloTag ligand and 2 μM antimicrobial peptides (either nisin or epilancin 15X). The solution was incubated in the dark at 37°C for 1 h, diluted with 500 μL of inner solution and analyzed with flow cytometry (BD LSR II). The fluorophore was excited with built-in blue laser and the emission monitored via Alexa Fluor 488 channel (530/30).

### 2.18. HaloTag cloning, expression and purification

pENTR4-HaloTag (w876-1) was a gift from Eric Campeau (Addgene plasmid #29644; RRID:Addgene_29,644).^1^ The HaloTag gene was amplified from plasmid pENTR4-HaloTag (w876-1) and inserted into pET28: His-MBP using Gibson assembly (NEB) to create pET28: His-MBP-TEV-HaloTag (Supplementary Table S2).

The plasmid encoding the His-tagged fusion protein was used to transform *E. coli* BL21 (DE3). Several colonies from overnight plates were used to inoculate 20 mL of TB media with 50 μg/mL kanamycin, cultured at 37°C for 5–6 h then sub-cultured in 500 mL of TB media with kanamycin at 37°C. When the OD_600_ reached 1–2, the culture was incubated on ice for 10 min, induced with 0.5 mM IPTG, and cultured at 18°C for 18–20 h. After harvesting, the cell pellet (~20 g/L culture) was resuspended in Start buffer (50 mM HEPES, 300 mM NaCl, 10 mM imidazole, pH 7.5) at ~3 mL/g pellet and stored at −70°C until use.

Frozen cell pellet from 0.5 L of culture was thawed and homogenized by passing three times through a French Press (Avestin C3) at 10,000–15,000 psi. The lysate was clarified by centrifugation at 25,000 ×g for 30 min. The supernatant was decanted into 1–2 mL Ni resin (Takara) pre-equilibrated in Start buffer. The mixture was rotated in a cold room for 30–45 min and loaded into an empty gravity column. The resin was washed with Wash buffer (50 mM HEPES, 300 mM NaCl, 50 mM imidazole, pH 7.5) until A_280_ < 0.05. Protein was eluted with 4 column volumes of Buffer B (50 mM HEPES, 300 mM NaCl, 300 mM imidazole, pH 7.5), then the buffer was exchanged to PBS buffer using a PD-10 column (Cytiva) and the solution concentrated to 600 μM using an Amicon 30 K MWCO.

## 3. Results

### 3.1. Epilancin 15X is bactericidal against *Staphylococcus carnosus* and *Bacillus subtilis*

**Figure 2:**
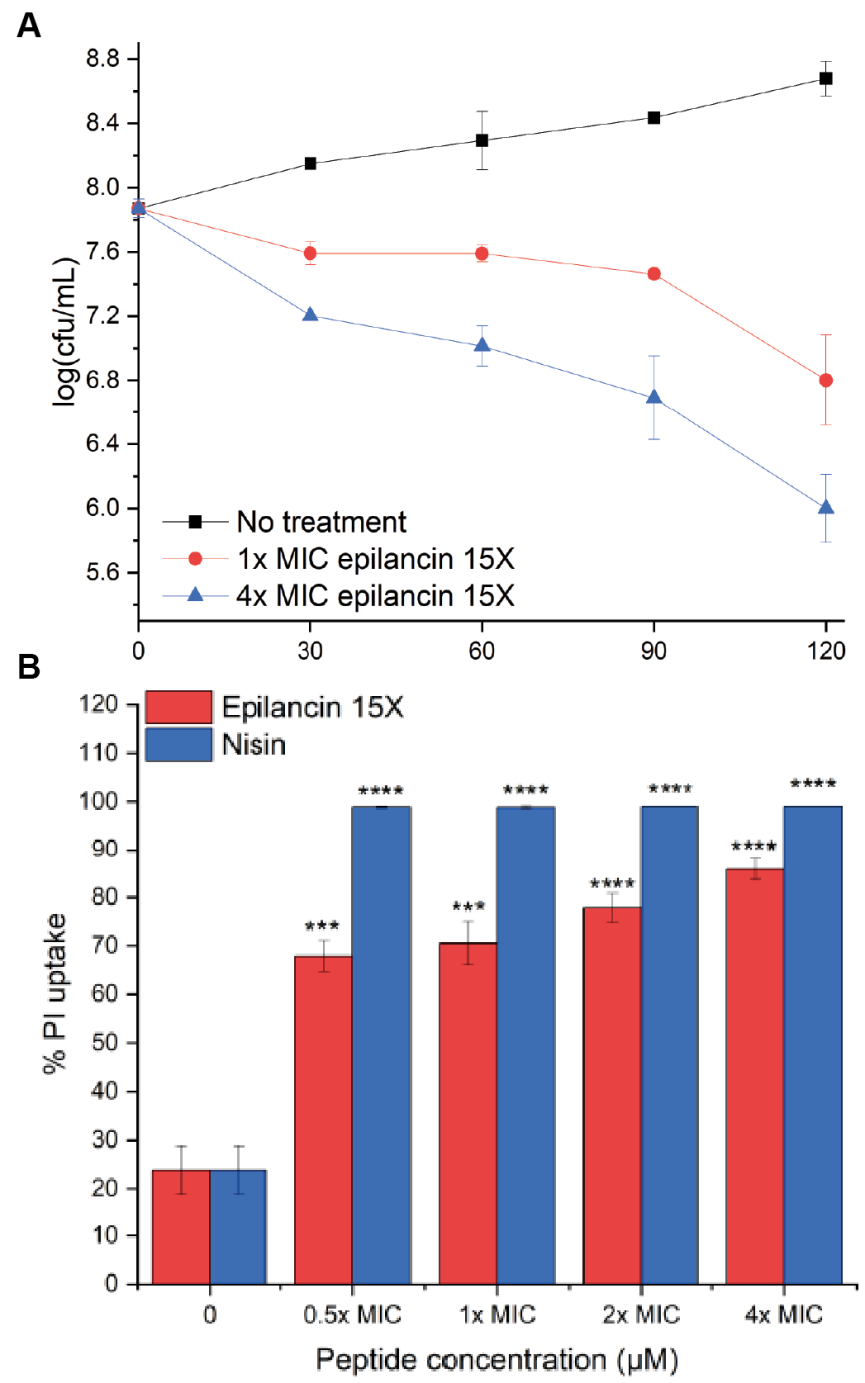
**(A)** Growth inhibition over time (min) of *Staphylococcus carnosus* TM300 by epilancin 15X. The data are representative of three independent experiments. Error bars indicate standard deviations. **(B)** Flow cytometry analysis of membrane disruption of *S. carnosus* TM300 by epilancin 15X. The results were calibrated such that the mean propidium iodide (PI) uptake from the 4x MIC nisin-treated cell culture was set as 100% PI uptake. The data are representative of three independent experiments. *** indicates a *p* value <0.001, and **** indicates a *p* value <0.0001 between peptide-treated cells and no treatment.

Epilancin 15X possesses a broad spectrum of antimicrobial activity against Gram-positive bacteria, including MICs in the sub-micromolar range against *Staphylococcus* strains (Table 1; Verhoef et al., 2005; Wang et al., 2023). Recent studies have shown that epilancin 15X activity varies depending on the indicator strain, with bacteriostatic activity against *Staphylococcus simulans* and *Micrococcus flavus* but bactericidal activity against *Bacillus megaterium* (Wang et al., 2023). In this study, *S. carnosus* TM300 and *B. subtilis* BSF2470 were investigated resulting in a significant decrease in the number of colony forming units (CFU), suggesting epilancin 15X exhibits bactericidal activity against these strains (Figure 2A; Supplementary Figure S1A).

**Table 1:** MIC values of epilancin 15X against selected bacterial strains.

Indicator strain	Epilancin 15X MIC (μM)
*S. carnosus* TM300	0.16
*S. epidermidis* ATCC 12228	0.62
*B. subtilis* 168	4.00
*B. subtilis* ATCC 6633	1.25
*B. subtilis* BSF2470 (1A980)	2.5

### 3.2. Epilancin 15X induces membrane disruption of *Staphylococcus carnosus* and *Bacillus subtilis* BSF2470

Epilancin 15X shares a similar C-terminal ring pattern with nisin (van de Kamp et al., 1995b; Ekkelenkamp et al., 2005), a known pore former (Hasper et al., 2004). Very recently, epilancin 15X was shown to disrupt the membrane of *B. megaterium,* toward which the compound had bactericidal activity, but not the membranes of *S. simulans* and *M. flavus,* toward which the lantibiotic had bacteriostatic activity (Wang et al., 2023). To investigate whether this correlation also holds for *S. carnosus* and *B. subtilis* BSF2470, we monitored the membrane integrity by examining propidium iodide (PI) uptake through flow cytometry. After 20 min exposure to epilancin 15X, an increase in PI uptake was observed (Figure 2B; Supplementary Figure S1B). The membrane disruption ability of the positive control nisin A was more substantial than that of epilancin 15X. The membrane potential was examined next by determining the red/green fluorescence ratio of the fluorescent dye 3,3′-diethyloxacarbocyanine (DiOC2; Novo et al., 1999). We observed an increase in the red/green fluorescence ratio with epilancin 15X treatment of *S. carnosus* (Supplementary Figure S2) indicating that epilancin 15X disrupts the membrane potential, consistent with its bactericidal activity.

### 3.3. Structure–activity relationship studies with epilancin 15X

Engineered variants of epilancin 15X or its structurally related cousins have not been reported, possibly because of the difficulty to genetically manipulate *S. epidermidis*. To investigate the SAR of epilancin 15X, in this work we performed substitutions of residues in the peptide with Ala residues using a recently developed heterologous expression system (Lee et al., 2023). Single residue substitution variants were generated using recombinant expression in *E. coli* BL21 (DE3) cells. Each His_6_-tagged mutant precursor peptide was post-translationally modified by ElxB and ElxC in *E. coli* with supplementation with the glutamyl-tRNA synthase (GluRS) and Glu-tRNA^Glu^ from *S. epidermidis*. The peptides were purified by immobilized metal ion affinity chromatography. After leader peptide removal by ElxP *in vitro* (Ortega et al., 2014), the peptides were further purified using semipreparative reversed-phase high-performance liquid chromatography (RP-HPLC). The purified peptides were characterized by matrix-assisted laser desorption/ionization time-of-flight mass spectrometry (MALDI-TOF MS; Supplementary Figure S1C).

To assess the effect of each mutation on the antibacterial activity, quantitative growth inhibition assays were performed in liquid culture against *S. carnosus* TM300 and compared to wild-type epilancin 15X. Individual alanine substitutions of positive-charged residues (K6A, K10A, K13A, K14A, K30A, K31A, R17A) revealed that all seven residues are important for antimicrobial activity (Table 2). This observation suggests a potential negatively charged binding partner(s) as the putative target of epilancin 15X. Alternatively or additionally, the alanine substitutions of these positively charged residues may negatively impact the overall structure of epilancin 15X. To probe whether epilancin activity was correlated with surface charge of bacteria, a cytochrome c binding assay was used to determine the relative surface charge of *S. carnosus* and *B. subtilis* (Supplementary Figure S2; Mukhopadhyay et al., 2007). No correlation between cytochrome c binding and epilancin MIC was observed suggesting that its antimicrobial activity is not a strong function of membrane charge and/or membrane binding is not the only factor important for antimicrobial activity.

**Table 2:** MIC values of epilancin 15X variants against *Staphylococcus carnosus* TM300.

Variants	MIC (μM)	Increase in MIC
WT epilancin 15X	0.16	N/A
ElxA_Lac1Pyr	0.16	None
ElxA_K6A	2.50	16X
ElxA_K10A	0.62	2X
ElxA_K13A	1.25	8X
ElxA_K14A	0.31	2X
ElxA_K30A	0.62	4X
ElxA_K31A	0.62	4X
ElxA_R17A	1.25	8X
ElxA_S3A, T7V, T8V	0.62	4X
ElxA_T28V	1.25	8X

N/A, not applicable.

The N-terminal dehydroamino acids (Dha3, Dhb7, Dhb8) and the Dhb near the C-terminus (Dhb28) also appeared to be important for the antimicrobial activity of epilancin 15X as replacement by Ala or Val resulted in increased MICs (Table 2). The decrease in bioactivity may be due to the loss of planarity of the unsaturated residues, which have been shown to induce conformational effects (Palmer et al., 1992). In contrast, the N-terminal Lac residue was not critical for bioactivity. Without adding ElxO dehydrogenase to the system (Velásquez et al., 2011), the peptide contains a pyruvate residue rather than a lactate residue on the N-terminus and this analog retained the same activity as the wild-type compound (Table 2).

### 3.4. Effect of epilancin 15X on macromolecular biosynthesis

**Figure 3:**
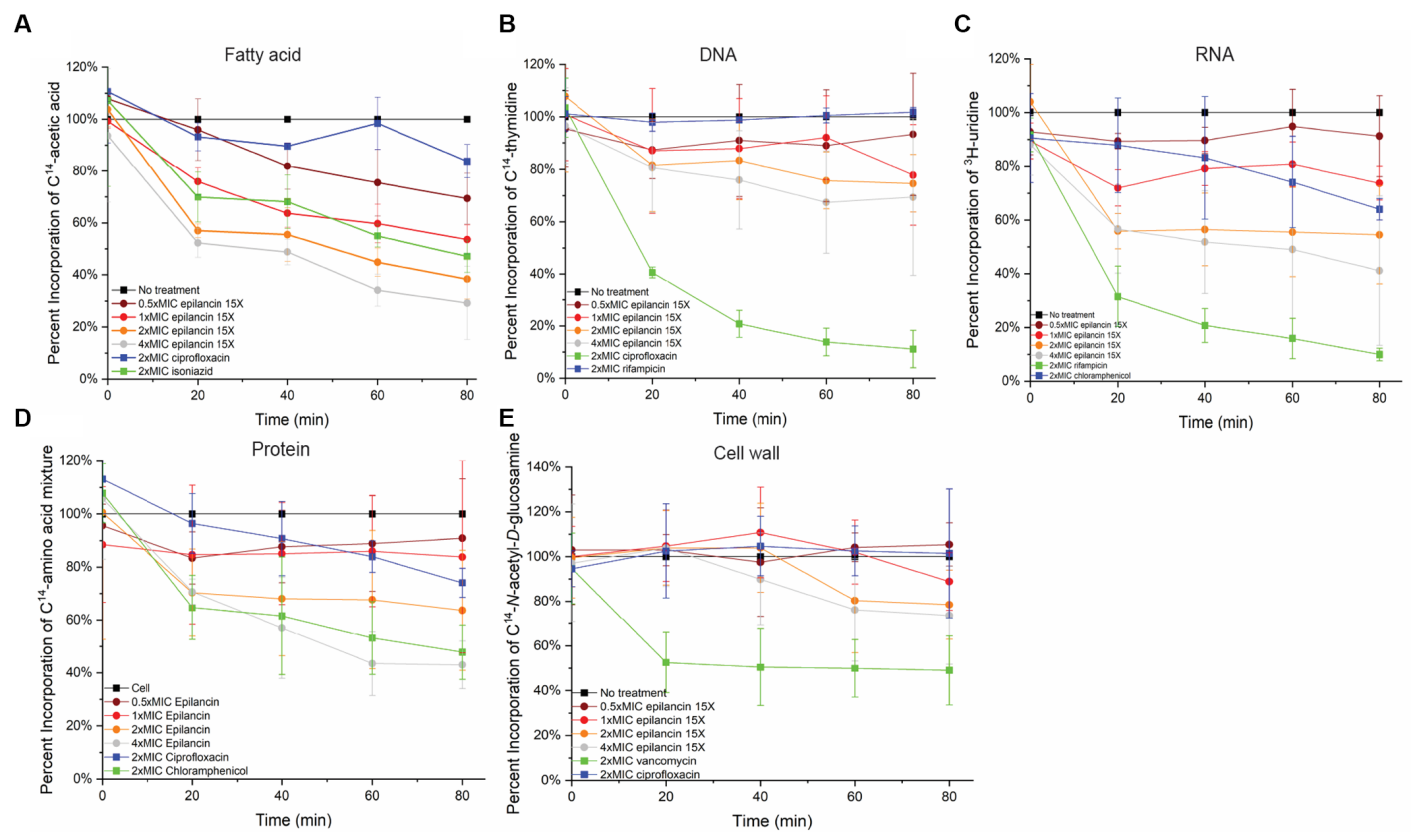
Epilancin 15X treatment results in the decrease of fatty acid, DNA, RNA, and protein biosynthesis without affecting cell wall synthesis. Time-dependent incorporation of **(A)** ^14^C-acetic acid into fatty acids, **(B)** ^14^C-thymidine into DNA, **(C)** ^3^H-uridine into RNA, **(D)** ^14^C-L-amino acids into protein, and **(E)** ^14^C-N-acetyl-D-glucosamine into cell wall in *S. carnosus* TM300. Positive controls (green) used were **(A)** isoniazid, **(B)** ciprofloxacin, **(C)** rifampicin, **(D)** chloramphenicol, and **(E)** vancomycin. Negative controls were untreated cell culture (black) as well as compounds with modes of action unrelated to the assay in question **(A)** ciprofloxacin, **(B)** rifampicin, **(C)** chloramphenicol, **(D,E)** ciprofloxacin (blue). The radioactivity of each antibiotic-treated culture was normalized based on its OD_600_ compared to an untreated cell culture. The percent incorporation was calibrated such that the radioactive precursor incorporation of the untreated cell culture was set as 100%. The data shown are from three independent experiments.

We next tested whether epilancin 15X has any effect on fatty acid, peptidoglycan, DNA, RNA, or protein biosynthesis in *S. carnosus* TM300 by examining the incorporation of radiolabeled precursors into these molecules compared to control samples. Epilancin 15X induced a decrease in fatty acid biosynthesis displaying a similar effect as that observed with the positive control isoniazid at the same relative concentration (2× MIC; Figure 3; Supplementary Figure S3A). In contrast, the disruption of radiolabel incorporation by the negative control ciprofloxacin was much smaller. In DNA, RNA, and protein biosynthesis pathways, epilancin 15X at 2× MIC displayed a considerably stronger effect than that observed with the respective negative controls. However, the positive controls affected these biosynthesis pathways more strongly than epilancin 15X (Figure 3; Supplementary Figures S3B–D).

In contrast, no clear change was observed in cell wall biosynthesis upon epilancin 15X treatment (Figure 3; Supplementary Figure S3E). The effect of epilancin 15X was comparable to the negative control ciprofloxacin, and it was significantly lower than the effect observed upon vancomycin treatment. These findings suggest that epilancin 15X does not inhibit the cell wall biosynthesis pathway and acts by a mechanism different from nisin.

### 3.5. Epilancin 15X does not induce LiaRS in *Bacillus subtilis* but upregulates VraRS in *Staphylococcus carnosus*

To investigate the effect of epilancin 15X on the cell envelope, we employed the two-component LiaRS reporter system in *B. subtilis* (lipid II cycle interfering antibiotic response regulator and sensor; Mascher et al., 2004). Briefly, the LiaRS assay is a disk diffusion assay used to screen for the ability of compounds to induce β-galactosidase expression in engineered *B. subtilis* BSF2470 (Mascher et al., 2004). Molecules that produce a blue ring around the edge of the zone of growth inhibition are believed to interfere with the lipid II cycle (Hachmann et al., 2009). Most membrane disrupting natural products that do not interfere with cell wall biosynthesis such as colistin, polymyxin, and monensin do not induce a LiaRS response (Wex et al., 2021). Bacitracin, vancomycin, and nisin are antibiotics that induce a LiaRS response as indicated by the blue ring at the edge of the zone of inhibition (Supplementary Figure S4A; Mascher et al., 2004). As expected, ampicillin did not induce a LiaRS response and neither did magainin 2 and melittin (Supplementary Figure S4A). The compound 1771, also called LtaS-IN-1, inhibits lipoteichoic acid (LTA) synthesis (Richter et al., 2013) and to the best of our knowledge has not been tested in the LiaRS assay. Unlike the wall teichoic acid (WTA) biosynthesis inhibitor tunicamycin that did result in a positive LiaRS response (Mascher et al., 2004), LtaS-IN-1 did not induce a blue zone, and epilancin 15X also did not induce a LiaRS response (Supplementary Figure S4A), thereby providing further support that epilancin 15X does not interfere with the cell wall biosynthesis pathway in *B. subtilis*.

Staphylococci have a similar system to LiaRS in *B. subtilis* called VraRS that responds to cell envelope stress (Kuroda et al., 2003; Utaida et al., 2003; Jordan et al., 2008). We did not have a reporter strain for *S. carnosus* but instead used RNAseq analysis. Upon treatment with sublethal amounts of epilancin 15X (0.5x MIC), expression of a number of genes was either up or downregulated (Supplementary Data file 1). Unexpectedly based on the LiaRS data in *B. subtilis*, expression of *vraR* and *vraS* was each upregulated 8-fold compared to untreated control and these genes were in the top 20 of upregulated genes (Supplementary Data file 1). As has been noted previously, although many Gram-positive bacteria possess systems that respond to cell envelope targeting compounds in similar ways, the stress responses appear genus or species specific (Jordan et al., 2008; McCallum et al., 2011).

### 3.6. Epilancin 15X localization by fluorescence microscopy

**Figure 4:**
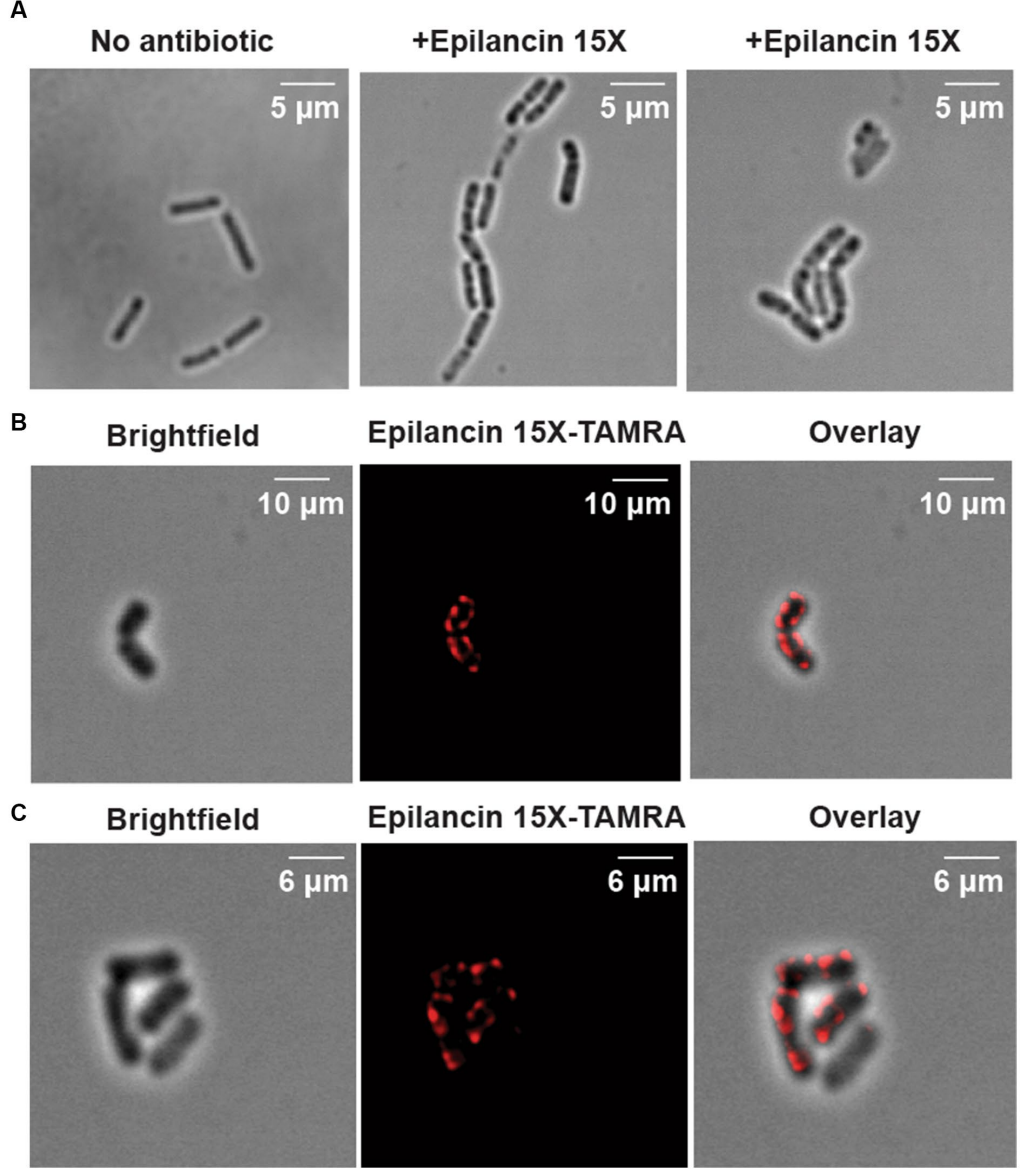
Super-resolution microscopy of *Bacillus subtilis* ATCC 6633 treated with epilancin 15X. **(A)** Representative brightfield images of *B. subtilis* ATCC 6633 treated with or without epilancin 15X. **(B,C)** Representative examples of epilancin 15X-TAMRA localization in *B. subtilis* ATCC 6633.

To gain further insights, we performed super-resolution microscopy studies to investigate the localization of epilancin 15X. First, the brightfield mode of Airyscan confocal microscopy was used to identify visual phenotypes of *B. subtilis* ATCC 6633 and *S. carnosus* TM300. Aggregation and chaining of cells was observed upon epilancin 15X treatment (Figure 4A; Supplementary Figure S4E). A similar aggregation effect has been reported when cells were treated with antimicrobial peptides such as melittin, complestatin, and corbomycin (Park et al., 2006; Culp et al., 2020). Melittin is a cationic antimicrobial peptide that interacts with membrane lipids and forms pores (Park et al., 2006). Complestatin and corbomycin are glycopeptides that block the action of autolysins, which are essential peptidoglycan hydrolases required for remodeling the cell wall during bacterial growth (Culp et al., 2020). Aggregation was not observed with nisin (Supplementary Figure S4F).

We prepared a fluorescently-labeled epilancin 15X analog by addition of TAMRA-thiol to the free Dha/Dhb of epilancin 15X. The epilancin 15X-TAMRA product was purified using HPLC and analyzed by MALDI-TOF MS (Supplementary Figure S4). Epilancin 15X-TAMRA displayed an MIC of 0.5 μM against *S. carnosus* TM300 (~3-fold MIC increase compared to wild-type epilancin 15X) and also induced the aggregation phenotype. Bacterial cells treated with epilancin 15X-TAMRA were fixed on slides and imaged by super-resolution Airyscan confocal laser-scanning microscopy. The images showed that epilancin 15X localized predominately at the surface of the bacteria in a punctate pattern (Figures 4B,C).

### 3.7. Lipid protection assays

The possibility of direct interaction between epilancin 15X and specific lipids was examined through lipid protection assays. Supplementing the growth medium with 2.5 μM LTA isolated from *S. aureus* resulted in the protection of *S. carnosus* TM300 from cell death by increasing the epilancin 15X MIC at least 8-fold (Supplementary Table S3). The protection by LTA was most effective, but other negatively-charged lipids, including 1-palmitoyl-2-oleoyl-sn-glycero-3-phospho-(1′-rac-glycerol; POPG) and cardiolipin were also able to increase the MIC at least 4-fold. Conversely, neutral lipids including 1-palmitoyl-2-oleoyl-sn-glycero-3-phosphocholine (POPC), 1-palmitoyl-2-oleoyl-sn-glycero-3-phosphoethanolamine (POPE), and digalactosyldiacylglycerol (DGDG) did not display any protection affect (Supplementary Table S3). In an agar diffusion assay, purified *S. aureus* LTA antagonized the bioactivity of epilancin 15X but not nisin A (Supplementary Figure S5A). These data suggest that negatively-charged lipids may be involved in the mode of action of epilancin 15X. Since epilancin 15X does not inhibit ^14^C-GlcNAc incorporation into macromolecules as shown in the radiolabeled precursor uptake experiment, we considered it unlikely that epilancin targets WTAs that consist of ribitol phosphate repeat units in which the ribitol residue is substituted with D-alanine and N-acetyl-D-glucosamine (GlcNAc; Pasquina et al., 2013).

### 3.8. Epilancin 15X induces leakage from liposomes containing negatively charged lipids

The previous results showed that epilancin 15X disrupts the integrity of the membrane of *S. carnosus* and *B. subtilis* and potentially interacts with negatively charged membrane lipids. We therefore investigated epilancin 15X-induced membrane disruption as a function of the lipid composition using a liposome leakage assay. Unilamellar liposomes were constructed with 1,2-dioleoyl-sn-glycero-3-phosphatidylcholine (DOPC) in the presence of various concentrations of supplemented lipids. We introduced three types of negatively charged membrane lipids [POPG, *S. aureus* LTA, and 1-palmitoyl-2-oleoyl-sn-glycero-3-phospho-L-serine (POPS)], and the neutral lipid POPE. Sulforhodamine-B (SRB) was encapsulated within lipid vesicles during liposome preparation. At high concentrations, this fluorophore exhibits self-quenching limiting the fluorescence emission. Upon membrane disruption, liposome leakage leads to the release of SRB into the medium and increased fluorescence emission.

In a 96-well plate, each well was loaded with liposome solution and antibiotics or the surfactant triton X-100 were added at 5 min. Fluorescence emission of SRB-loaded liposomes was monitored and dye leakage was not detected when DOPC liposomes were treated with epilancin 15X up to 64 μM. With the incorporation of negatively charged membrane lipids such as LTA, POPS, and POPG in the DOPC liposomes, the fluorescence emission from the dye leakage increased as the concentration of epilancin 15X increased (Supplementary Figures S5B–D). However, dye leakage was not detected when DOPC liposomes were doped with neutral membrane lipids such as POPE (Supplementary Figure S5E).

**Figure 5:**
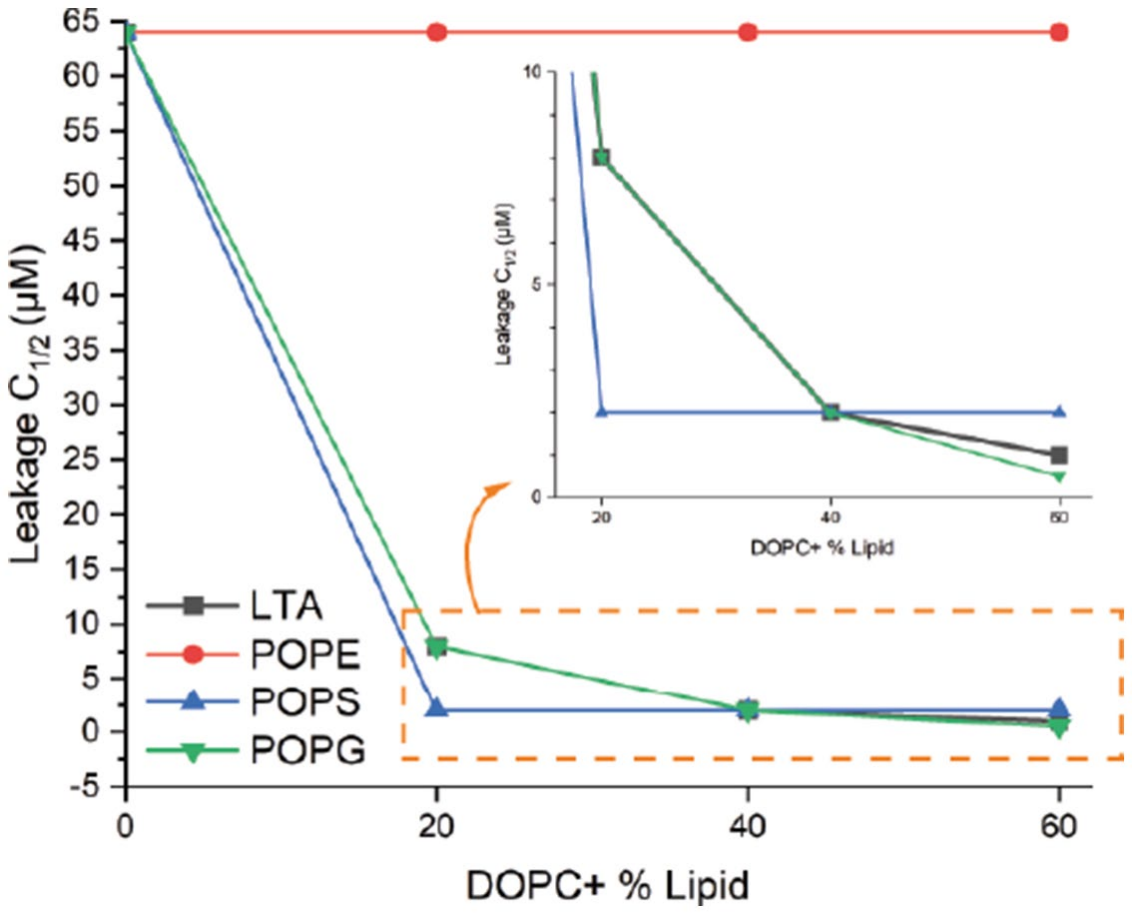
Liposome permeabilization assay. Epilancin 15X C_1/2_ values as a function of supplemented lipid concentration. For each of the supplemental lipid concentrations, the concentration of epilancin 15X where liposome leakage reached 50% of the positive control triton X-100 leakage was measured as C_1/2_. Four lipids with various concentrations were tested: *S. aureus* LTA, POPE, POPS, and POPG.

The percent leakage of each liposome was calibrated according to the fluorescence emission of triton X-100 as 100% leakage (Supplementary Figures S6A–C). For each of the supplemental lipid concentrations, the concentration of epilancin 15X where liposome leakage reached 50% of the triton X-100 leakage was measured as C_1/2_ (Supplementary Figures S6A–C). The epilancin 15X C_1/2_ values were plotted against the supplemented lipid concentrations (Figure 5). If the epilancin 15X induced liposome leakage depends on the supplemented lipid concentration, a diagram with a negative slope should be observed (van den Bogaart et al., 2008). As shown in Figure 5, epilancin 15X induced liposome leakage with a negative slope of C_1/2_ values when lipids carry negative charges, including *S. aureus* LTA, POPG, and POPS. In contrast, no change in liposome leakage was observed with neutral lipids, including POPC and POPE. In summary, epilancin 15X can form pores in liposomes containing negatively charged lipids and not neutral lipids. The degree of pore formation correlates with the negatively charged lipid concentration.

As shown above, epilancin 15X does not result in a LiaRS response in *B. subtilis* but does result in upregulation of VraRS in *S. carnosus*. Furthermore, lipid II was recently shown to antagonize the activity of the compound (Wang et al., 2020), and the mechanism of action of epilancin 15X has been suggested to be different depending on the bacteria investigated (Wang et al., 2023). Therefore, to assess lipid II binding in a culture-independent manner, we also examined the ability of epilancin 15X to permeabilize liposomes containing lipid II. POPC-cholesterol liposomes containing 0.1% lipid II were constructed and tested. The liposomes encapsulated the HaloTag protein whereas the Alexa Fluor 488 (AF488) labeled HaloTag ligand was introduced to the surrounding solution along with the tested antimicrobial peptide (Supplementary Figure S6D). Upon pore formation, the HaloTag substrate was anticipated to be able to enter the liposomes and form a covalent linkage with the HaloTag-containing protein, which prevents the efflux of the ligand due to the size of the protein, resulting in the accumulation of fluorescent signal inside the liposomes (Supplementary Figure S6D). The liposome population was then examined using flow cytometry. Compared to the buffer control, no increase in fluorescence signal was detected when POPC-cholesterol liposomes were treated with epilancin 15X or nisin (Supplementary Figure S6E). When 0.1% lipid II was incorporated into POPC-cholesterol liposomes, a substantial increase in fluorescence signal was observed upon treatment with 1 μM nisin, indicating pore-forming activity that was lipid II dependent (Supplementary Figure S6F). In contrast, 1 μM epilancin 15X did not induce pore formation on the same liposomes (Supplementary Figure S6F). This result is consistent with the observation that epilancin 15X does not inhibit ^14^C-N-acetyl-D-glucosamine incorporation into the cell wall in *S. carnosus* and does not induce a LiaRS response in *B. subtilis*.

## 4. Discussion

Epilancin 15X is a bacteriocin isolated from *S. epidermidis* 15×154. It is a member of the larger epilancin-group of lanthipeptides that has potent antimicrobial activity against several pathogenic bacteria with one of the lowest MIC values among reported lanthipeptides against *Staphylococcus* strains. Epilancin 15X treatment of *S. carnosus* affects fatty acid synthesis as strongly as the positive control isoniazid. DNA replication and transcription, and RNA translation are also affected, but these effects are likely downstream effects because they were not as strong as the positive controls. The incorporation of cell wall biosynthesis precursors was not altered by epilancin 15X. Thus, while the fatty acid biosynthesis pathway cannot be ruled out, other macromolecular synthesis pathways do not appear to be a direct target. Interestingly, daptomycin was previously shown to result in membrane detachment of the phospholipid synthase PlsX (Müller et al., 2016). PlsX catalyzes conversion of acyl-ACP to acyl-phosphate during phosphatidic acid biosynthesis and its inhibition directly affects fatty acid synthesis (Paoletti et al., 2007). Thus, similar PlsX displacement could explain the observed phenotype of reduced fatty acid biosynthesis upon epilancin 15X treatment. Future studies will evaluate this possibility.

This study demonstrates that epilancin 15X is bactericidal against *S. carnosus* and *B. subtilis* and disrupts their membranes. SAR studies indicate that the positively charged residues and Dha/Dhb residues are important for the bioactivity of epilancin 15X, consistent with protection and membrane disruption experiments that suggest a negatively charged binding partner or a non-specific affinity for negatively charged membrane components. As discussed in the introduction, a model has been proposed in which a series of positively charged residues surrounding a thioether ring that are found in many non-lipid II targeting lantibiotics may be important for antimicrobial activity. In support of this proposal, we observed large increases in MIC upon mutation of Lys13 and Arg17, with a smaller effect upon mutation of Lys10 and an additional strong effect for mutation of Lys6. The localization of fluorescently labeled epilancin 15X on the surface of the bacteria is also consistent with a mechanism that involves membrane lipids.

Unlike other membrane-active antibiotics such as nisin and daptomycin (Hachmann et al., 2009), epilancin 15X did not induce a LiaRS response in *B. subtilis*. However, RNAseq analysis in *S. carnosus* TM300, which is considerably more sensitive to epilancin 15X than the *B. subtilis* strains tested (Table 1), showed that VraSR expression was upregulated. These differences in response are not unlike a recent other study that showed differences of distinct bacterial strains toward epilancin 15X (Wang et al., 2023). The different LiaRS and VraSR responses leave unresolved the possibility that epilancin acts on the cell envelope in a manner beyond membrane disruption, but we do not think it likely that epilancin interacts directly with the cell wall precursor lipid II given the preponderance of evidence against this possibility in the current and previous studies (liposome assays, transglycosylation assays, binding assays, *N-*AcGlcNAc incorporation into cell wall; Brötz et al., 1998; Oman et al., 2011). But we cannot rule out interference with autolysis given the chaining phenotype observed. Compounds that inhibit autolysins have been shown to induce strong *ywaC* responses in *B. subtilis* (Culp et al., 2020), a cell envelope stress system akin to *liaRS*, which is not seen with treatment with epilancin 15X. Whether or not a specific negatively charged membrane lipid is responsible for the low MICs against especially staphylococci is at present not clear and requires further investigation.

## Data availability statement

The original contributions presented in the study are included in the article/Supplementary material, further inquiries can be directed to the corresponding author.

## Author contributions

CW, BL, RM, DD, and TL collected the data. YS conducted RNAseq analysis. The manuscript was written by CW and WD. All authors contributed to the article and approved the submitted version.

## Footnotes

^1^ http://n2t.net/addgene:29644

## Glossary

RiPPs: ribosomally synthesized and post-translationally modified peptides
MRSA: methicillin resistant *Staphylococcus aureus*
VRE: vancomycin resistant Enterococcus sp.
Lan: lanthionine
MeLan: methyllanthionine
Dha: dehydroalanine
Dhb: dehydrobutyrine
Abu: 2-aminobutyric acid
SAR: structure–activity relationship
RP-HPLC: reversed-phase high-performance liquid chromatography
MALDI-TOF MS: matrix-assisted laser desorption/ionization time-of-flight mass spectrometry
GluRS: glutamyl-tRNA synthase
LB: Luria-Bertani broth
TB: terrific broth
BHI: brain heart infusion
HBS: HEPES-Buffered Saline
TFA: trifluoroacetic acid
DiOC2: 3,3′-diethyloxacarbocyanine
PI: Propidium iodide
SRB: sulforhodamine B
MIC: minimal inhibitory concentration
CAMP: cationic antimicrobial peptides
TA: teichoic acids
LTA: lipoteichoic acid
WTA: wall teichoic acids
POPC: 1,2-Dioleoyl-sn-glycero-3-phosphocholine
POPC: 1-palmitoyl-2-oleoyl-sn-glycero-3-phosphocholine
POPE: 1-palmitoyl-2-oleoyl-sn-glycero-3-phosphoethanolamine
POPG: 1-palmitoyl-2-oleoyl-sn-glycero-3-phospho-(1′-rac-glycerol)
POPS: 1-palmitoyl-2-oleoyl-sn-glycero-3-phospho-L-serine
DOPC: 1,2-dioleoyl-sn-glycero-3-phosphocholine
DGDG: digalactosyldiacylglycerol
PDI: polydispersity index
DLS: dynamic light scattering
ΜMP: UDP-MurNAc-pentapeptide
GlcNAc: N-acetylglucosamine
MurNAc: N-Acetylmuramic acid
IPA: isopropyl alcohol
IPTG: Isopropyl β-D-1-thiogalactopyranoside
MOPS: 3-(N-morpholino)propanesulfonic acid
PBS: phosphate-buffered saline
