# Supplementary material for: Investigation into the mechanism of action of the antimicrobial peptide epilancin 15X
Source: Front Microbiol. 2023 Nov 2;14:1247222. doi: 10.3389/fmicb.2023.1247222 (PMC10652874; doi:10.3389/fmicb.2023.1247222)
Supplement: Supplementary file 1 [file Data_Sheet_1.zip › Figure_S1.PDF]

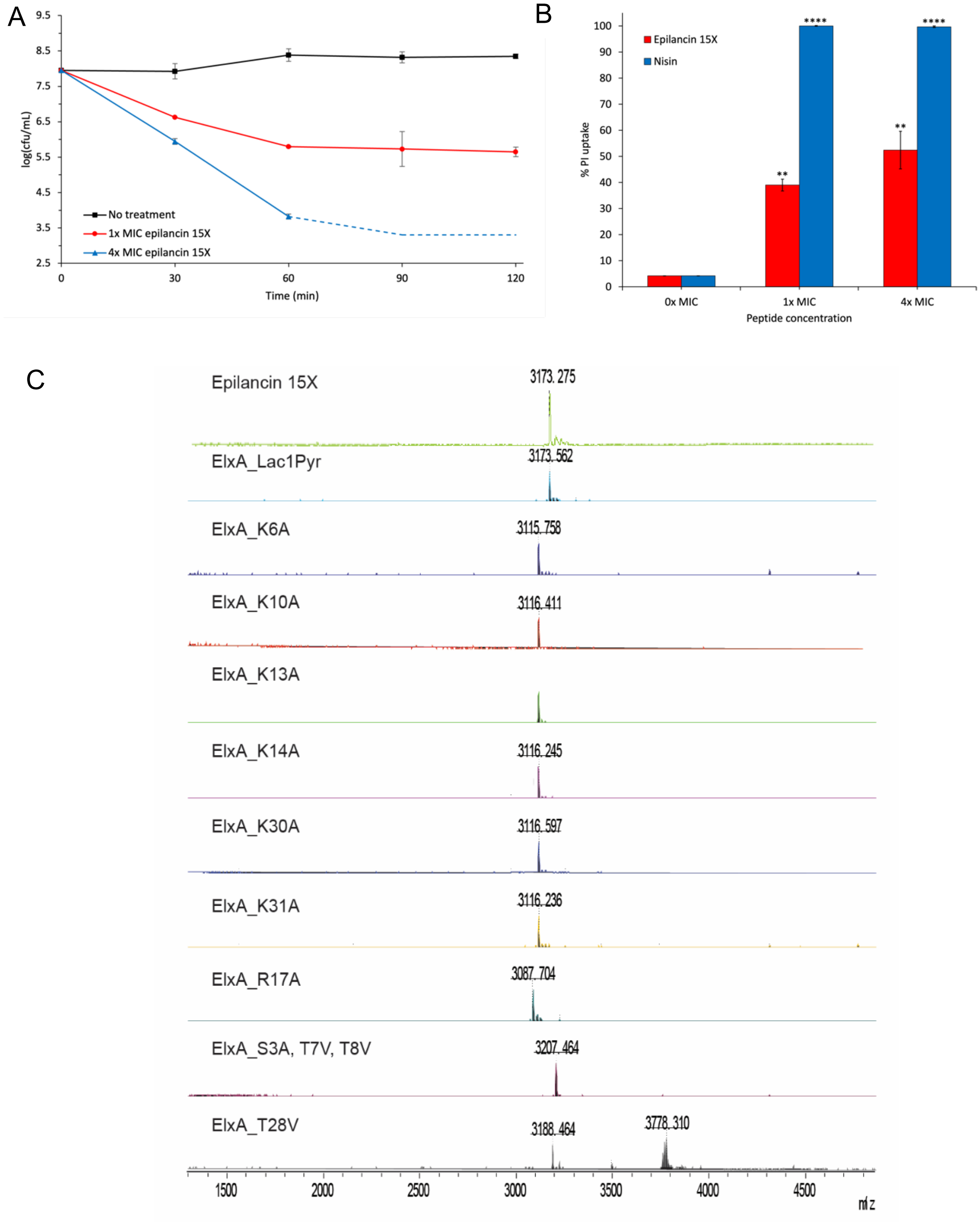

Figure S1. (A) Growth inhibition of *B. subtilis* BSF2470 by epilancin 15X. The data are representative of three independent experiments. Error bars indicate standard deviation, error bars are smaller than marker if not observed. The dashed line for 4x MIC epilancin 15X treatment is a projection of the lower limit of growth (1 colony) since no growth was observed for 90 and 120 minutes at the dilution factor plated. (B) Flow cytometry analysis of membrane disruption of *B. subtilis* BSF2470 by epilancin 15X. The results were calibrated such that the mean propidium iodide (PI) uptake from the 4x MIC nisin-treated cell culture was set as 100% PI uptake. The data are representative of three independent experiments. ** indicates a P-value < 0.01, and **** indicates a P-value < 0.0001 between peptide-

treated cells and no treatment. (C) MALDI-TOF MS analysis of epilancin 15X analogs that were isolated from the epilancin 15X co-expression system with ElxBC and the pEVOL platform in *E. coli* after leader peptide removal by ElxP and purification with RT-HPLC. Calculated m/z values: Epilancin 15X & ElxA_Lac1Pyr: 3172; ElxA_K6A, K10A, K13A, K14A and K30A: 3114; ElxA_R17A: 3086.
