# Supplementary material for: Investigation into the mechanism of action of the antimicrobial peptide epilancin 15X
Source: Front Microbiol. 2023 Nov 2;14:1247222. doi: 10.3389/fmicb.2023.1247222 (PMC10652874; doi:10.3389/fmicb.2023.1247222)
Supplement: Supplementary file 1 [file Data_Sheet_1.zip › Figure_S2.PDF]

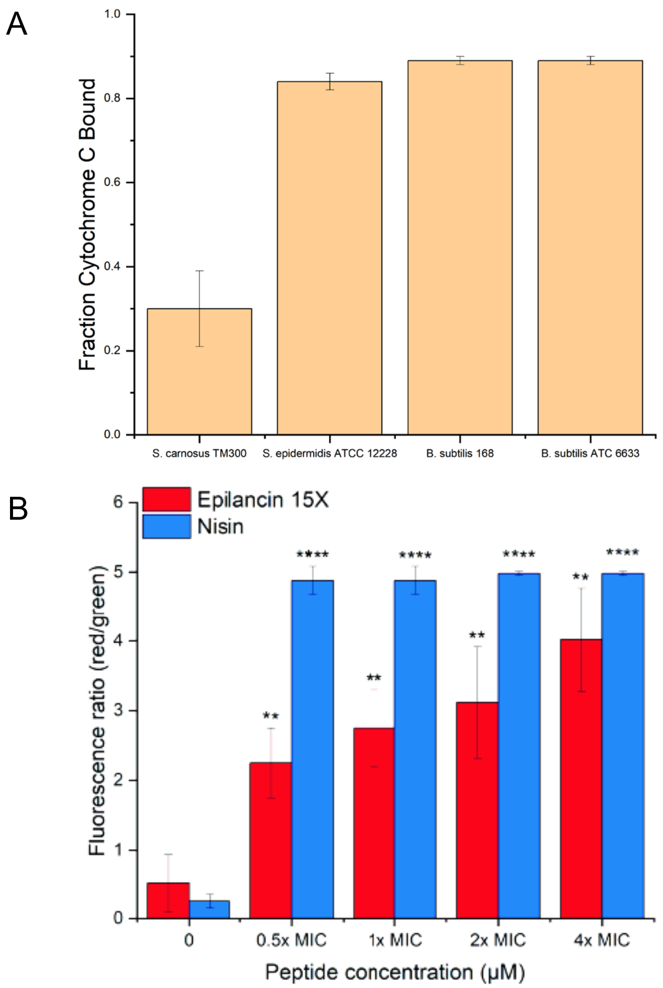

Figure S2. (A) Binding of the cationic protein cytochrome C to whole cells of four different bacterial strains. Fraction bound was determined by comparing the absorbance at 530 nm of a cell treated sample with a cell-free blank. Error bars represent the standard deviation of three separate trials. (B) Flow cytometry analysis of the membrane disruption by epilancin 15X in *S. carnosus* TM300. Membrane depolarization activity was measured by the mean red/green fluorescence ratio using DiOC2. The data are representative of three independent experiments. ** indicates a P-value < 0.01, *** indicates a P-value < 0.001, and **** indicates a P-value < 0.0001 between peptide-treated cells and no treatment.
