# Supplementary material for: Investigation into the mechanism of action of the antimicrobial peptide epilancin 15X
Source: Front Microbiol. 2023 Nov 2;14:1247222. doi: 10.3389/fmicb.2023.1247222 (PMC10652874; doi:10.3389/fmicb.2023.1247222)
Supplement: Supplementary file 1 [file Data_Sheet_1.zip › Figure_S3.PDF]

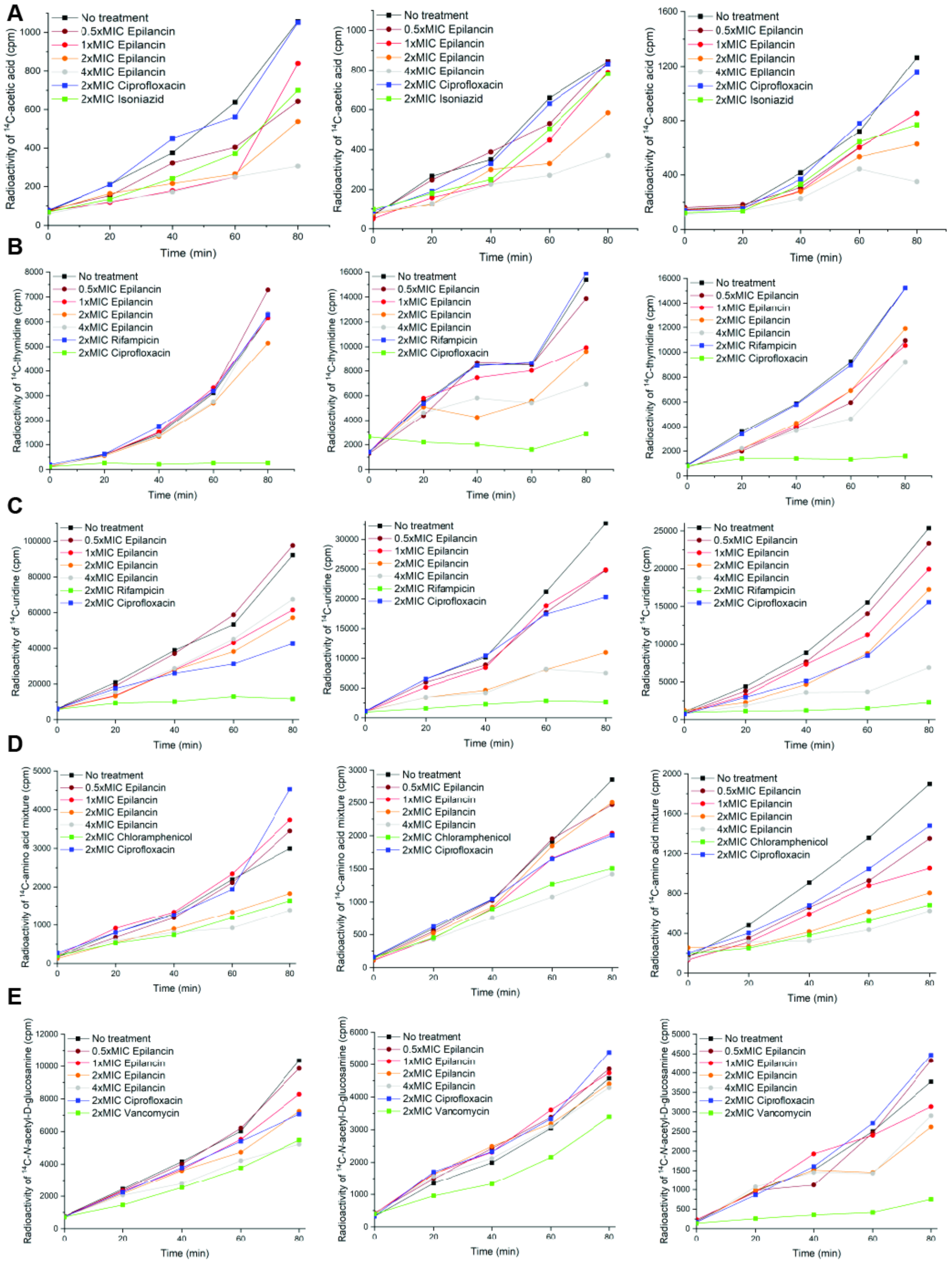

Figure S3. Three independent experiments of incorporation of (A) ^{14}C-acetic acid into fatty acids, (B) ^{14}C-thymidine into DNA, (C) ^{3}H-uridine into RNA, (D) ^{14}C-labeled amino acids into protein, and (E) ^{14}C-labeled GlcNAc into cell wall in *S. carnosus* TM300 cells treated with various antimicrobial agents. The percent incorporation at 0 min was measured after incubating the cell culture with 0.8 μCi/mL radioactive precursors for 15 min. Then 0.5x, 1x, 2x, or 4x MIC epilancin 15X and 2x MIC controls were added to the cell culture (t=0), and the percent incorporation was measured over time. Positive controls are green and negative controls are black (cells only) and blue (compound with mode of action unrelated to the assay in question). The radioactivity of each antibiotic-treated culture was normalized based on its OD_{600} at each time point compared to the untreated cell culture.
