# Supplementary material for: Investigation into the mechanism of action of the antimicrobial peptide epilancin 15X
Source: Front Microbiol. 2023 Nov 2;14:1247222. doi: 10.3389/fmicb.2023.1247222 (PMC10652874; doi:10.3389/fmicb.2023.1247222)
Supplement: Supplementary file 1 [file Data_Sheet_1.zip › Figure_S4.PDF]

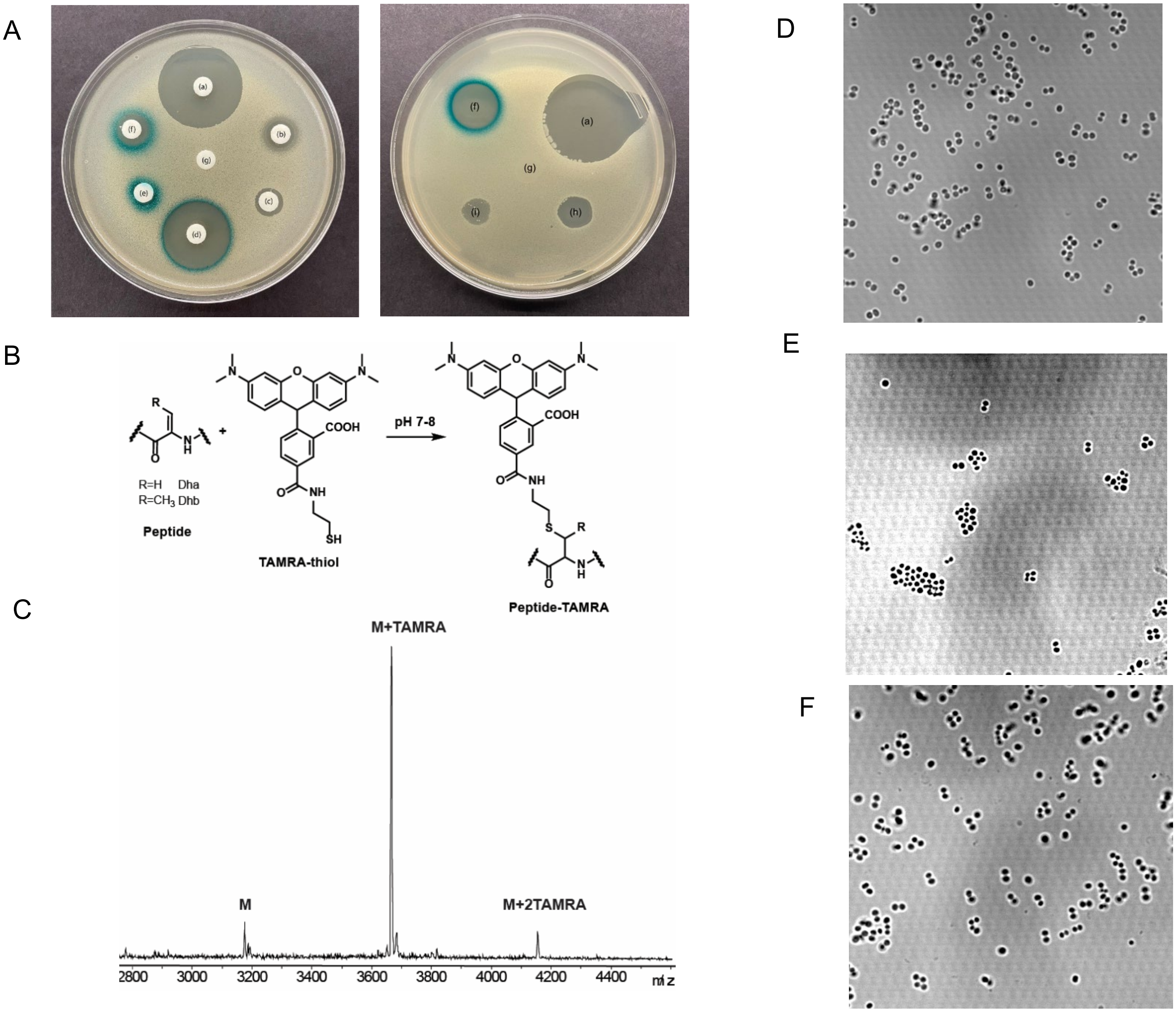

Figure S4. (A) LiaRS disk diffusion assay to determine lipid II cycle interfering antibiotics. The following molecules were tested to screen their ability to induce β-galactosidase activity in strain *B. subtilis* BSF2470: (a) ampicillin, (b) compound 1771 (LtaS-IN-1), (c) epilancin 15X, (d) vancomycin, (e) nisin, (f) bacitracin, (g) water, (h) melittin, and (i) magainin 2. A blue ring around the edge of the zone of inhibition indicates the molecule induces a LiaRS response and interferes with the lipid II cycle. (B) Synthesis of epilancin 15X-TAMRA using TAMRA-thiol. By addition to Dha/Dhb containing peptides. (C) MALDI-TOF MS spectrum of HPLC purified epilancin 15X-TAMRA. Expected [M+TAMRA]: 3664.54, observed: 3664.43. Expected [M+2TAMRA]: 4156.15, observed: 4153.69. Given the much higher reactivity of Dha compared to Dhb, the TAMRA group is most likely attached at position 3 of epilancin 15X. (D) Bright field image of *S. carnosus* TM300. (E) Bright field image of *S. carnosus* treated with 0.5 MIC epilancin 15X (88 nM). (F) Bright field image of *S. carnosus* TM300 treated with 0.5 MIC nisin (250 nM).
