# Supplementary material for: Investigation into the mechanism of action of the antimicrobial peptide epilancin 15X
Source: Front Microbiol. 2023 Nov 2;14:1247222. doi: 10.3389/fmicb.2023.1247222 (PMC10652874; doi:10.3389/fmicb.2023.1247222)
Supplement: Supplementary file 1 [file Data_Sheet_1.zip › Figure_S5.PDF]

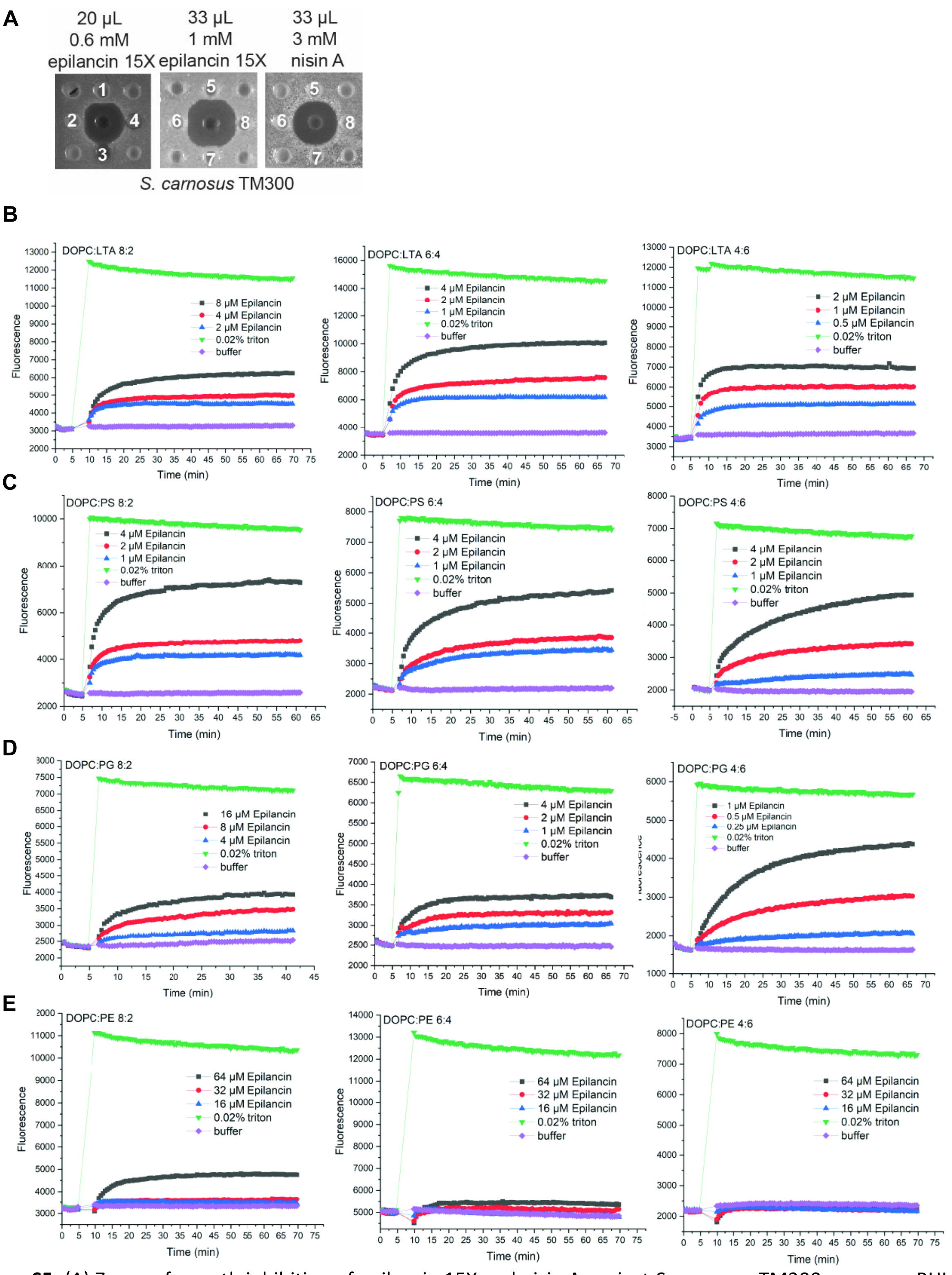

Figure S5. (A) Zones of growth inhibition of epilancin 15X and nisin A against *S. carnosus* TM300 grown on BHI agar plates. The indicated amounts of antibiotics were placed in the well shown in the center of the zone of inhibition. A total of 20 µL lipids at various concentrations were added to the labeled wells flanking the central well: (1) 10 mM *S. aureus* LTA; (2) 10 mM *B. subtilis* LTA; (3) 10 mM DGDG; (4) 10 mM POPE; (5) 5 mM *S. aureus* LTA; (6) 2.5 mM *S. aureus* LTA; (7) 1.25 mM *S. aureus* LTA; and (8) 20 µL water. All lipids were diluted in water and the zone of inhibition was examined after overnight incubation of bacterial cells with antibiotics and lipids. (B-E) Liposome

permeabilization as a function of time. Fluorescence emission upon adding epilancin 15X to liposomes supplemented with various concentrations of *S. aureus* LTA (B), POPS (C), POPG (D), and POPE (E). Triton X-100 was added as a positive control, and buffer was added as a negative control.
