# Supplementary material for: Investigation into the mechanism of action of the antimicrobial peptide epilancin 15X
Source: Front Microbiol. 2023 Nov 2;14:1247222. doi: 10.3389/fmicb.2023.1247222 (PMC10652874; doi:10.3389/fmicb.2023.1247222)
Supplement: Supplementary file 1 [file Data_Sheet_1.zip › Figure_S6.PDF]

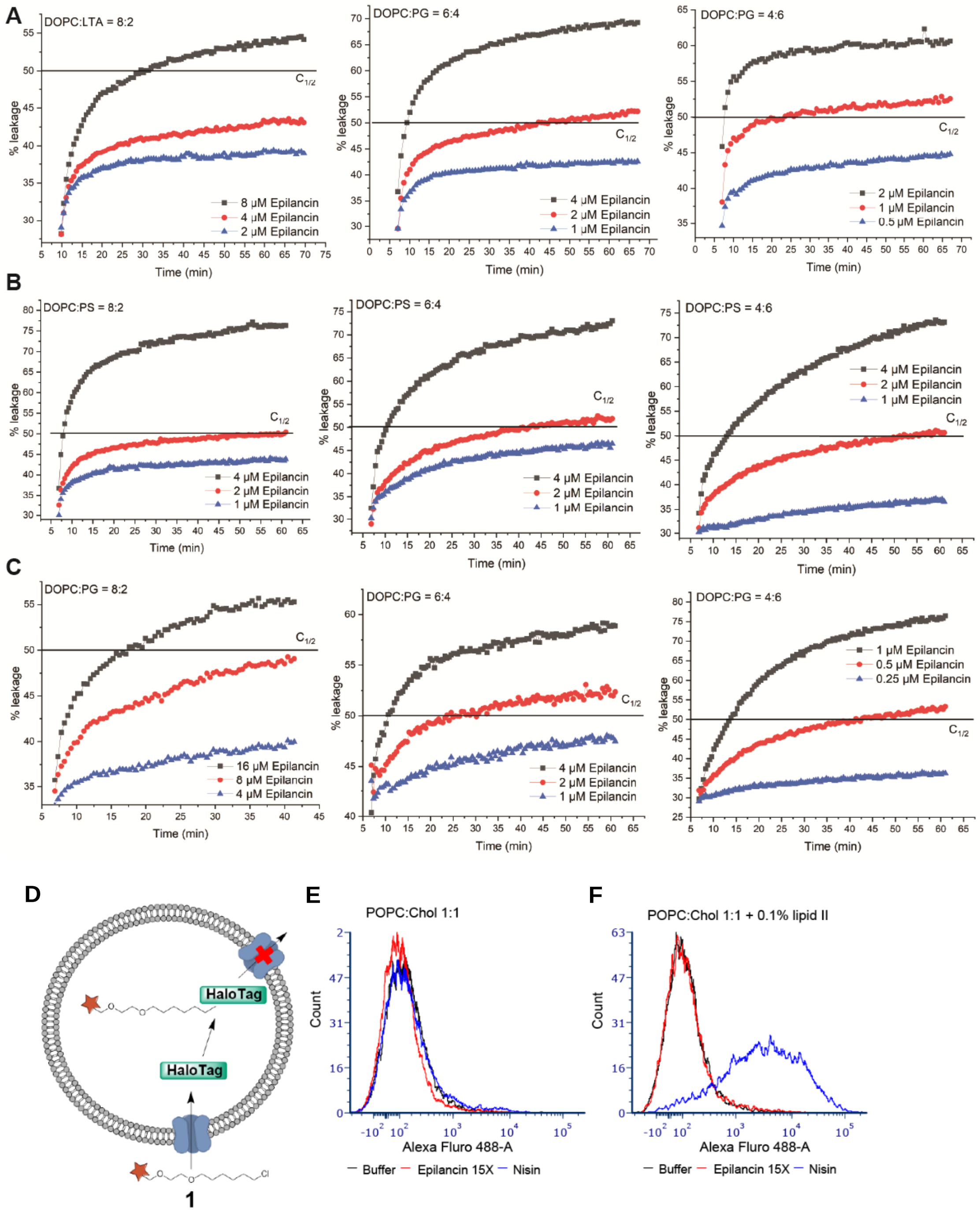

Figure S6. Liposome permeabilization as a function of time. DOPC liposomes made after supplementation with one of three lipids at various concentrations were used: *S. aureus* LTA (A), POPS (B), and POPG (C). The percent permeabilization was normalized such that the fluorescence emission of the triton X-100 treated sample was 100%. The concentration of epilancin 15X that induced 50% leakage at equilibrium is shown as $C_{1/2}$. The percent leakage of liposomes made of POPE is not shown because 50% permeabilization was not reached with the highest concentration (64 μM) of epilancin 15X tested. (D-F) Liposome permeabilization assay of liposomes containing lipid II. (D) Schematic diagram of AF488-labelled substrate 1 entering liposomes upon pore formation and being covalently linked to a HaloTag protein. (E,F) Fluorescence counts are shown of flow cytometry of liposomes composed of POPC:cholesterol (1:1) with (E) or without (F) 0.1% lipid II after treatment with 1 μM epilancin 15X and

AF488-labelled peptide 1. Untreated liposomes were used as the negative control and 1 µM nisin was used as the positive control.
