# Supplementary material for: Investigation into the mechanism of action of the antimicrobial peptide epilancin 15X
Source: Front Microbiol. 2023 Nov 2;14:1247222. doi: 10.3389/fmicb.2023.1247222 (PMC10652874; doi:10.3389/fmicb.2023.1247222)
Supplement: Supplementary file 1 [file Data_Sheet_1.zip › Table_S1.PDF]

Table S1. Strains and plasmids used in this study.

Strains or plasmids	Purpose	Source or reference
**Strains**		
E. coli BL21(DE3)	Heterologous expression strain	NEB
E. coli Turbo	Plasmid propagation strain	NEB
Staphylococcus epidermidis 15X154	Epilancin 15X producer strain	(Ekkelenkamp et al., 2005)
Staphylococcus carnosus TM300	Epilancin 15X sensitive strain	G. Bierbaum, U. of Bonn
Bacillus subtilis non-essential gene knockout library	Screening for potential target pathway of epilancin 15X	Addgene kit 1000000115
**Plasmids**		
pET21a_His_6ElxA	Overexpression of ElxA and mutants in *E. coli*	(Lee et al., 2023)
pRSFDuet_ElxC_ElxB	Overexpression of ElxB and ElxC in *E. coli*	(Lee et al., 2023)
pEVOL_GluRS_GluRS_$tRNA^{Glu}$	Overexpression of S. epidermidis 15X GluRS and $tRNA^{Glu}$ under various promoters in *E. coli*	(Lee et al., 2023)
pET28_MurG-His_6	Glycosyltransferase that adds GlcNAc onto lipid I to form lipid II	This study
pTrc33_ His_{10}HyMraY	Translocase that adds MurNac-peptapeptide onto phosphorylated undecaprenol to form lipid I	This study

References

Ekkelenkamp, M.B., Hanssen, M., Hsu, S.T., de Jong, A., Milatovic, D., Verhoef, J., et al. (2005). Isolation and structural characterization of epilancin 15X, a novel lantibiotic from a clinical strain of *Staphylococcus epidermidis*. *FEBS Lett.* 579(9), 1917-1922. doi: 10.1016/j.febslet.2005.01.083.

Lee, H., Wu, C., Desormeaux, E.K., Sarksian, R. and van der Donk, W.A. (2023). Improved production of class I lanthipeptides in Escherichia coli. *Chem. Sci.* doi: 10.1039/D2SC06597E.
