# Supplementary material for: Investigation into the mechanism of action of the antimicrobial peptide epilancin 15X
Source: Front Microbiol. 2023 Nov 2;14:1247222. doi: 10.3389/fmicb.2023.1247222 (PMC10652874; doi:10.3389/fmicb.2023.1247222)
Supplement: Supplementary file 1 [file Data_Sheet_1.zip › Table_S2.PDF]

Table S2. Primers, gene fragments, and protein sequences used in this study.

**Primers**	
MurG_F	tgtttaactttaagaaggagatataccatgagtggtcaaggaaagcgattaatg
MurGHis_R	tcagtggtggtggtggtggccgctgctgcccgcccgggcaacccggctc
pET28-CHis-bb_R	catggtatatctccttcttaaagttaaaca
pET28-CHis-bb_F	ggcagcagcggccaccaccaccaccac
SARgBlock BB_F	cccttggggcctctaaac
SARgBlock BB_R	acaattcccctatagtgagtcg
SARgBlock Insert_F	actcactataggggaattgtgagcggataacaattccc
SARgBlock Insert_R	ccgtttagaggccccaaggggttatgctagttattgctcag
**Gene Fragments**	
ElxA _K6A	gagcggataacaattccccatcttagtatattagttaagtataagaaggagatatacatATGGGCAGCAGCCATCACCATCATCACCACAGCCAGGATCCGATGAAAAAAGAATTATTTGATTTAAATCTTAATAAAGATATCGAGGCACAAAAAAGTGACCTAAATCCGCAATCAGCTAGTATTGTTGCGACAACTATCAAAGCTTCTAAAAAGCTTTGTAGAGGATTTACACTAACTTGTGGATGTCACTTTACTGGTAAAAAATAAttaattaacctaggctgctgccaccgctgagcaataactagcataac
ElxA _K10A	gagcggataacaattccccatcttagtatattagttaagtataagaaggagatatacatATGGGCAGCAGCCATCACCATCATCACCACAGCCAGGATCCGATGAAAAAAGAATTATTTGATTTAAATCTTAATAAAGATATCGAGGCACAAAAAAGTGACCTAAATCCGCAATCAGCTAGTATTGTTAAAACAACTATCGCGGCTTCTAAAAAGCTTTGTAGAGGATTTACACTAACTTGTGGATGTCACTTTACTGGTAAAAAATAAttaattaacctaggctgctgccaccgctgagcaataactagcataac
ElxA _K13A	gagcggataacaattccccatcttagtatattagttaagtataagaaggagatatacatATGGGCAGCAGCCATCACCATCATCACCACAGCCAGGATCCGATGAAAAAAGAATTATTTGATTTAAATCTTAATAAAGATATCGAGGCACAAAAAAGTGACCTAAATCCGCAATCAGCTAGTATTGTTAAAACAACTATCAAAGCTTCTGCGAAGCTTTGTAGAGGATTTACACTAACTTGTGGATGTCACTTTACTGGTAAAAAATAAttaattaacctaggctgctgccaccgctgagcaataactagcataac

**Gene Fragments (continued)**	
ElxA _K14A	gagcggataacaattccccatcttagtatattagttaagtataagaaggagatatacatATGGGCAGCAGCCATCACC<br>ATCATCACCACAGCCAGGATCCGATGAAAAAAGAATTATTTGATTTAAATCTTAATAAAGATATCGAGGCACAAAAAA<br>GTGACCTAAATCCGCAATCAGCTAGTATTGTTAAAACAACTATCAAAGCTTCTAAAGCGCTTTGTAGAGGATTTACAC<br>TAACTTGTGGATGTCACTTTACTGGTAAAAAATAAttaattaacctaggctgctgccaccgctgagcaataactagca<br>taac
ElxA _K30A	gagcggataacaattccccatcttagtatattagttaagtataagaaggagatatacatATGGGCAGCAGCCATCACC<br>ATCATCACCACAGCCAGGATCCGATGAAAAAAGAATTATTTGATTTAAATCTTAATAAAGATATCGAGGCACAAAAAA<br>GTGACCTAAATCCGCAATCAGCTAGTATTGTTAAAACAACTATCAAAGCTTCTAAAAAGCTTTGTAGAGGATTTACAC<br>TAACTTGTGGATGTCACTTTACTGGTGCGAAATAAttaattaacctaggctgctgccaccgctgagcaataactagca<br>taac
ElxA _K31A	gagcggataacaattccccatcttagtatattagttaagtataagaaggagatatacatATGGGCAGCAGCCATCACC<br>ATCATCACCACAGCCAGGATCCGATGAAAAAAGAATTATTTGATTTAAATCTTAATAAAGATATCGAGGCACAAAAAA<br>GTGACCTAAATCCGCAATCAGCTAGTATTGTTAAAACAACTATCAAAGCTTCTAAAAAGCTTTGTAGAGGATTTACAC<br>TAACTTGTGGATGTCACTTTACTGGTAAAGCGTAAttaattaacctaggctgctgccaccgctgagcaataactagca<br>taac
ElxA _R17A	gagcggataacaattccccatcttagtatattagttaagtataagaaggagatatacatATGGGCAGCAGCCATCACC<br>ATCATCACCACAGCCAGGATCCGATGAAAAAAGAATTATTTGATTTAAATCTTAATAAAGATATCGAGGCACAAAAAA<br>GTGACCTAAATCCGCAATCAGCTAGTATTGTTAAAACAACTATCAAAGCTTCTAAAAAGCTTTGTGCGGGATTTACAC<br>TAACTTGTGGATGTCACTTTACTGGTAAAAAATAAttaattaacctaggctgctgccaccgctgagcaataactagca<br>taac
ElxA _S3A,T7V, T8V	gagcggataacaattccccatcttagtatattagttaagtataagaaggagatatacatATGGGCAGCAGCCATCACC<br>ATCATCACCACAGCCAGGATCCGATGAAAAAAGAATTATTTGATTTAAATCTTAATAAAGATATCGAGGCACAAAAAA<br>GTGACCTAAATCCGCAATCAGCTGCGATTGTTAAAGTGGTGATCAAAGCTTCTAAAAAGCTTTGTAGAGGATTTACAC<br>TAACTTGTGGATGTCACTTTACTGGTAAAAAATAAttaattaacctaggctgctgccaccgctgagcaataactagca<br>taac
ElxA _T28V	gagcggataacaattccccatcttagtatattagttaagtataagaaggagatatacatATGGGCAGCAGCCATCACC<br>ATCATCACCACAGCCAGGATCCGATGAAAAAAGAATTATTTGATTTAAATCTTAATAAAGATATCGAGGCACAAAAAA<br>GTGACCTAAATCCGCAATCAGCTAGTATTGTTAAAACAACTATCAAAGCTTCTAAAAAGCTTTGTAGAGGATTTACAC<br>TAACTTGTGGATGTCACTTTGTGGGTAAAAAATAAttaattaacctaggctgctgccaccgctgagcaataactagca<br>taac

His-MBP-TEV-HaloTag	MGSSHHHHHHSSGLVPRGSHMKIEEGKLVIWINGDKGYNGLAEVGK<br>KFEKDTGIKVTVEHPDKLEEKFPQVAATGDGPDIIFWAHDRFGGYA<br>QSGLLAEITPDKAFQDKLYPFTWDAVRYNGKLIAYPIAVEALSLIY<br>NKDLLPNPPKTWEEIPALDKELKAKGKSALMFNLQEPYFTWPLIAA

	DGGYAFKYENGKYDIKDVGVDNAGAKAGLTFLVDLIKNKHMNADTD<br>YSIAEAAFNKGETAMTINGPWAWSNIDTSKVNYGVTVLPTFKGQPS<br>KPFVGVLSAGINAASPNKELAKEFLENYLLTDEGLEAVNKDKPLGA<br>VALKSYEEELAKDPRIAATMENAQKGEIMPNIPQMSAFWYAVRTAV<br>INAASGRQTVDEALKDAQTNSSSHHHHHHANSVPLVPRGSENLYFQ<br>SMAEIGTGFPFDPHYVEVLGERMHYVDVGPRDGTPVLFLHGNPTSS<br>YVWRNIIPHVAPTHRCIAPDLIGMGKSDKPDLGYFFDDHVRFMDAF<br>IEALGLEEVVLVIHDWGSALGFHWAKRNPERVKGIAFMEFIRPIPT<br>WDEWPEFARETFQAFRTTDVGRKLIIDQNVFIEGTLPMGVVRPLTE<br>VEMDHYREPFLNPVDREPLWRFPNELPIAGEPANIVALVEEYMDWL<br>HQSPVPKLLFWGTPGVLIPPAEAARLAKSLPNCKAVDIGPGLNLLQ<br>EDNPDLIGSEIARWLSTLEISG*
MurG-CHis	MSGQGKRLMVMAGGTGGHVFPGLAVAHHLMAQGWQVRWLGTADRME<br>ADLVPKHGIEIDFIRISGLRGKGIKALIAAPLRIFNAWRQARAIMK<br>AYKPDVVLGMGGYVSGPGGLAAWSLGIPVVLHEQNGIAGLTNKWLA<br>KIATKVMQAFPGAFPNAEVVGNPVRTDVLALPLPQQRLAGREGPVR<br>VLVVGGSQGARILNQTMPQVAAKLGDSVTIWHQSGKGSQQSVEQAY<br>AEAGQPQHKVTEFIDDMAAAYAWADVVVCRSGALTVSEIAAAGLPA<br>LFVPFQHKDRQQYWNALPLEKAGAAKIIEQPQLSVDAVANTLAGWS<br>RETLLTMAERARAASIPDATERVANEVSRVARAGSSGHHHHHH*
His_{10}-HyMraY	MGSSHHHHHHHHHHENLYFQGMIYHLAILLREHFFAFNVLKYITFR<br>SFTAILLAFFITLILSPTFMKKFAKIQRLFGGYVREYTPEHHESKK<br>YTPTMGGVVIVTVILITSVLLMRLDIRYTWVLVFSTLSFALIGFVD<br>DWIKLKNKKGLSIKAKLAFQMSFALAVSLLIFYWVGLETKLYFPFF<br>KELTVDLGWLYIPFSMFIIVGTANAVNLTDGLDGLAIGPSMTTATA<br>FGVIAYVVGHSKIAQYLGVPHVPYAGEITVFCFAIIGAGLGFLWFN<br>TYPAQVFMGDVGALGLGAALATVSIMTKSEFLLAVAGGVFVFETVT<br>VILQIIYFRATGGKRLFRKAPFHHHLEEKGLDEPKIVVRMWIVSAL<br>LAIVSVAMLKLR*
