# Supplementary material for: Investigation into the mechanism of action of the antimicrobial peptide epilancin 15X
Source: Front Microbiol. 2023 Nov 2;14:1247222. doi: 10.3389/fmicb.2023.1247222 (PMC10652874; doi:10.3389/fmicb.2023.1247222)
Supplement: Supplementary file 1 [file Data_Sheet_1.zip › Table_S3.PDF]

Table S3. Protection effects of lipids against epilancin 15X activity on *S. carnosus* TM300.

Compound added (charge)	Final concentration (µM)	Epilancin MIC (µM)	Increase in MIC compared to WT
POPG (-)	100	1.25	4X
POPE	500	0.31	No change
DGDG	100	0.31	No change
POPC	130	0.31	No change
Cardiolipin (-)	130	2.5	8X
B. subtilis LTA (-)	~100	2.5	8X
S. aureus LTA (-)	~100	>5	>16X
